# Probiotic *Bacillus subtilis* Protects against α-Synuclein Aggregation in *C. elegans*

**DOI:** 10.1016/j.celrep.2019.12.078

**Published:** 2020-01-14

**Authors:** María Eugenia Goya, Feng Xue, Cristina Sampedro-Torres-Quevedo, Sofia Arnaouteli, Lourdes Riquelme-Dominguez, Andrés Romanowski, Jack Brydon, Kathryn L. Ball, Nicola R. Stanley-Wall, Maria Doitsidou

**Affiliations:** 1 University of Edinburgh, Centre for Discovery Brain Sciences, Edinburgh, Scotland; 2 University of Dundee, School of Life Sciences, Dundee, Scotland; 3 University of Edinburgh, School of Biological Sciences, Edinburgh, Scotland; 4 University of Edinburgh, Institute of Genetics & Molecular Medicine, Edinburgh, Scotland

**Keywords:** probiotics, *B. subtilis*, *C. elegans*, α-synuclein, microbiota, Parkinson’s disease, DAF-16/FOXO, dietary restriction, sphingolipid metabolism, biofilm

## Abstract

Recent discoveries have implicated the gut microbiome in the progression and severity of Parkinson’s disease; however, how gut bacteria affect such neurodegenerative disorders remains unclear. Here, we report that the *Bacillus subtilis* probiotic strain PXN21 inhibits α-synuclein aggregation and clears preformed aggregates in an established *Caenorhabditis elegans* model of synucleinopathy. This protection is seen in young and aging animals and is partly mediated by DAF-16. Multiple *B. subtilis* strains trigger the protective effect via both spores and vegetative cells, partly due to a biofilm formation in the gut of the worms and the release of bacterial metabolites. We identify several host metabolic pathways differentially regulated in response to probiotic exposure, including sphingolipid metabolism. We further demonstrate functional roles of the sphingolipid metabolism genes *lagr-1*, *asm-3*, and *sptl-3* in the anti-aggregation effect. Our findings provide a basis for exploring the disease-modifying potential of *B. subtilis* as a dietary supplement.

## Highlights

• *B. subtilis* PXN21 inhibits and reverses α-syn aggregation in a *C. elegans* model
• Spores and vegetative cells protect through different mechanisms
• The probiotic inhibits α-syn aggregation by changing the host sphingolipid metabolism
• Biofilm formation in the gut and bacterial metabolites reduce α-syn aggregation

## Graphical Abstract

**Figure:**
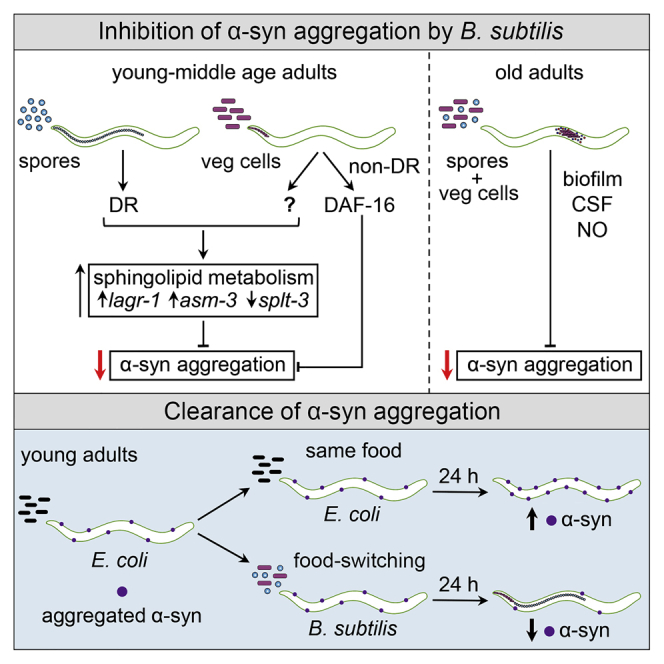

## Teaser

How the gut microbiome affects Parkinson’s disease remains unclear. Goya et al. show that the probiotic *B. subtilis* strain PXN21 inhibits and clears α-synuclein aggregation in a *C. elegans* model. The bacterium acts via metabolites and biofilm formation to activate protective pathways in the host, including DAF-16/FOXO and sphingolipid metabolism.

## Introduction

Protein misfolding and aggregation are key pathological features observed in numerous neurodegenerative diseases, including Alzheimer’s and Parkinson’s disease (PD) (Ross and Poirier, 2004). PD is one of the most prevalent neurodegenerative disorders (Pringsheim et al., 2014) and is currently incurable. It is characterized by the progressive loss of dopaminergic neurons in the Substantia Nigra area of the brain, leading to the development of progressive motor and non-motor symptoms (Poewe et al., 2017). Central to the condition is the accumulation of α-synuclein (α-syn) aggregates in Lewy bodies (Spillantini et al., 1998), and the extent of this accumulation correlates with disease severity (Stefanis, 2012). α-syn acquires neurotoxic properties when protein monomers progressively combine to form insoluble amyloid fibrils via oligomeric intermediates (Poewe et al., 2017). Although Lewy bodies contain mostly fibrillar forms of α-syn, oligomeric intermediates are also toxic and play a central role in PD pathogenesis (Winner et al., 2011). Despite recent progress toward identifying disease-modifying interventions (Savitt and Jankovic, 2019), only symptomatic treatments are available (Fahn, 2015). Thus, therapeutic strategies directed at inhibiting or reversing α-syn aggregation present a clear opportunity for disease-modifying interventions for PD and other synucleinopathies.

Although PD is primarily considered to be a central nervous system disease, there is clear evidence for an involvement of peripheral signals, particularly from the gastrointestinal tract and the gut microbiota, in PD progression. This is supported by observations that PD symptoms and α-syn pathology begin in peripheral tissues, particularly the intestine, and as the disease progresses, α-syn aggregates gradually spread to multiple brain regions (Braak et al., 2003, Rietdijk et al., 2017). Recently, the human gut microbiome has emerged as an important player influencing PD (Scheperjans, 2016). Gut bacteria can affect brain function by producing metabolites that enter the bloodstream, eliciting immune responses in the host or modulating neuronal function (Chow et al., 2010, Fung et al., 2017). Preclinical evidence suggests that the gut microbiota and intestinal permeability modulate behavior, mood, and neuropsychiatric disorders (Clapp et al., 2017). Likewise, a large number of recent studies investigating microbiota in patients with PD found notable differences compared to healthy controls (reviewed by Boertien et al., 2019), which correlated with clinical features (Li et al., 2017, Minato et al., 2017, Scheperjans et al., 2015). Remarkably, faecal transplants from PD patients exacerbate symptoms in a mouse model of PD, demonstrating that differences in microbiota are not merely a result of the disease, but also impact its progression (Sampson et al., 2016).

Human microbiota consist of trillions of microorganisms and over 1,000 bacterial species (Lloyd-Price et al., 2016), posing a challenge for understanding the effects of individual species. In the bacterivore *Caenorhabditis elegans*, the gut microbiota can be precisely controlled, making it a powerful model for studying the effects of gut bacteria on physiological processes at a single species-single gene level (Cabreiro and Gems, 2013). Furthermore, *C. elegans* has proven to be a valuable model for studying molecular mechanisms of PD and protein aggregation. Overexpression of human α-syn in *C. elegans* results in the formation of aggregates that progressively become amyloid-like (Kaminski Schierle et al., 2011, van Ham et al., 2008), and work in *C. elegans* models has identified conserved genetic and chemical modifiers of α-syn toxicity (Büttner et al., 2013, Hamamichi et al., 2008, Kautu et al., 2013, Knight et al., 2014, Kuwahara et al., 2008, Pujols et al., 2018, Qiao et al., 2008, Roodveldt et al., 2009, Ruan et al., 2010, van Ham et al., 2008, Zhang et al., 2017). Here, we used a *C. elegans* model of synucleinopathy to investigate the effects of gut bacteria on α-syn aggregation.

We report that the probiotic bacterium *Bacillus subtilis* PXN21 (Colenutt and Cutting, 2014), when fed to *C. elegans*, inhibits, delays, and reverses α-syn aggregation. We characterize these protective effects in both young and old nematodes and investigate the contributions of known lifespan-extending pathways. We further show that *B. subtilis* extracts are able to partially recapitulate the protective effect of live bacteria, indicating that a bacterial metabolite is actively involved. From analysis of gene expression profiles, we find that the protective effect of *B. subtilis* against α-syn aggregation is mediated through alterations in the sphingolipid metabolism pathway. Our findings contribute to the current understanding of how gut bacteria interact with the host to influence physiology in remote tissues, and they will motivate further explorations of the probiotic *B. subtilis* as a diet-based intervention for PD.

## Results

### *B. subtilis* Inhibits and Reverses α-Syn Aggregation in a *C. elegans* Model of Synucleinopathy

To assess the effect of gut bacteria on α-syn aggregation, we used an established *C. elegans* model (strain NL5901), expressing human α-syn fused to yellow fluorescent protein (YFP) and driven by a muscle-specific promoter (P*unc-54*::α-syn::YFP) (van Ham et al., 2008). We fed these worms with different bacterial diets and assessed α-syn aggregation in day 1 adult animals (72 h post hatching). Among the bacterial species tested was the *B. subtilis* strain PXN21 (Colenutt and Cutting, 2014), isolated from the commercially available probiotic product Bio-Kult (by ADM Protexin).

**Figure 1:**
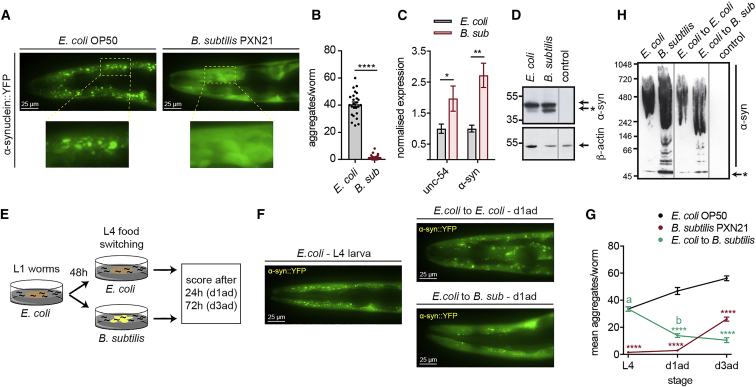
*B. subtilis* PXN21 Inhibits and Reverses α-Syn Aggregation in the *C. elegans* Model NL5901 (P*unc-54*::α-syn::YFP) (A) Representative fluorescent images of α-syn aggregates (foci) in the head of day 1 adult worms fed on *E. coli* OP50 or *B. subtilis* PXN21. Higher magnifications of the highlighted regions are shown. (B) Quantification of α-syn aggregates larger than 1 μm^2^ per animal in the head region of day 1 adult worms fed on the indicated diet. ^∗∗∗∗^p < 0.0001; n = 25 worms per condition. (C) Expression levels by qRT-PCR of *unc-54* and α-syn transcripts in day 1 adult worms normalized to the *E. coli* diet. Expression level of each gene in worms fed with *E. coli* was taken as 1. ^∗^p = 0.0245, ^∗∗^p = 0.0029, n = 3 per condition, with three technical replicates each (N represents a population of ∼4,000 worms). (D) SDS-PAGE of α-syn transgenic and wild-type (control column) day 1 adult worms grown on the two diets. Arrow and arrow with ^∗^ indicate α-syn monomeric and sub-monomeric forms, respectively. (E) Assay strategy for the food-switch experiment. L1, first larval stage; L4, fourth larval stage; d1ad, adult day 1; d3ad, adult day 3. (F) Fluorescent images of α-syn aggregates of representative L4 (left) and day 1 adult (upper right) worms grown on *E. coli* or 24 h after the switch to *B. subtilis* diet (lower right). (G) Average number of α-syn aggregates before and after the worm switching. ^∗∗∗∗^p < 0.0001 versus *E. coli*; a versus b, ^∗∗∗∗^p < 0.0001; n = 25 worms per time point per condition. (H) Immunoblotting of native α-syn conformations of transgenic and wild-type young adult worms. Arrow with ^∗^ indicates α-syn sub-monomeric form. Data shown are mean ± SEM from one representative experiment out of three with similar results.

On a regular *C. elegans* laboratory diet, comprising the non-pathogenic strain of *Escherichia coli* OP50 (Brenner, 1974), α-syn-expressing animals formed aggregates that can be visualized by fluorescence microscopy (van Ham et al., 2008) (Figures 1A and 1B). In contrast, animals fed on *B. subtilis* strain PXN21 showed a nearly complete absence of aggregates at the day 1 adult stage (Figures 1A and 1B). This striking difference in aggregation was not caused by lower expression levels of α-syn in PXN21-fed animals, as *unc-54* and α-syn transcript levels were upregulated in day 1 adult animals fed with *B. subtilis* (Figure 1C). Consistently, there were higher levels of α-syn protein in animals fed on the probiotic (Figures 1D and S1A).

We next tested whether a *B. subtilis* diet could also clear already-formed aggregates. We grew nematodes on *E. coli* until the fourth larval (L4) stage when aggregates are evident, then shifted them to a *B. subtilis* PXN21 diet (Figure 1E) and quantified α-syn aggregation 1 and 3 days later. Most of the aggregates present at the L4 stage cleared 1 day after switching diets, whereas the average size of the foci remained unaffected (Figures 1F, 1G, S1B, and S1C). The clearance of aggregates was not due to reduced levels of α-syn expression (Figures S1D–S1F). Notably, the reduced aggregation levels after the switch to *B. subtilis* persisted for longer, compared to animals grown continuously on this diet from the first larval (L1) stage (Figure 1G). Similar results were obtained in experiments where the food switch happened on the first day of adulthood (Figures S1G and S1H). We further investigated α-syn native forms using non-denaturing gel electrophoresis. Whereas high molecular mass α-syn forms were detected in extracts from worms ingesting either diet, lower molecular weight species that go down to a submonomeric form were primarily detected with the *B. subtilis* diet (Figure 1H). This indicates alterations of α-syn forms, possibly through cleavage or degradation, by this dietary condition.

### *B. subtilis* Protection Is Effective throughout *C. elegans* Aging

To assess the effects of a *B. subtilis* diet on α-syn aggregation in aging, we followed animals fed on *E. coli* OP50 or *B. subtilis* PXN21 until day 13 of adulthood (corresponding to day 16 of the worm’s life). We assessed aging animals under two different feeding conditions: (1) grown continuously on the specified diet for their entire life or (2) grown on *E. coli* until the L4 stage and then shifted to a *B. subtilis* diet.

**Figure 2:**
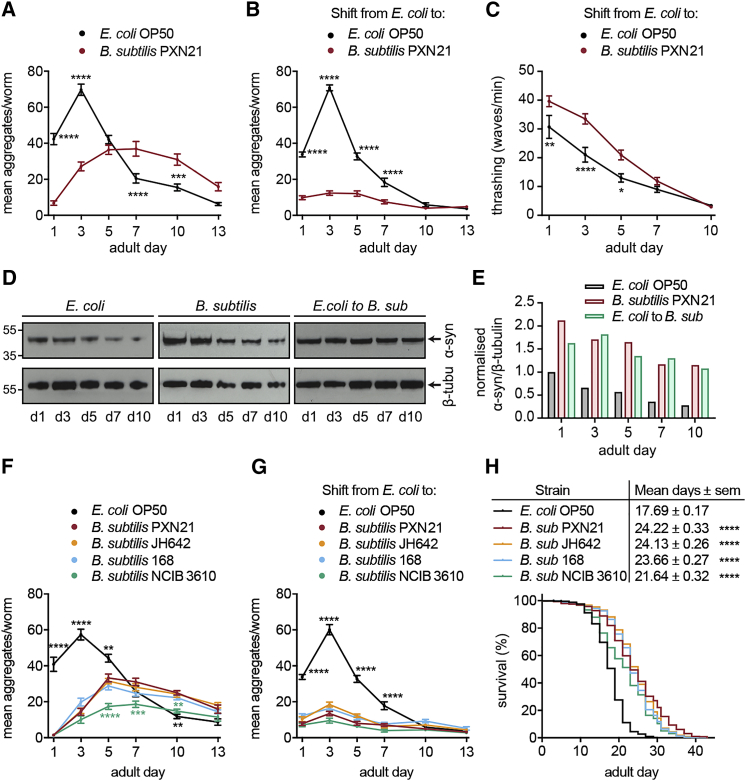
*B. subtilis* Protection against α-Syn Aggregation Is Effective throughout *C. elegans* Aging and Is Triggered by Different Strains (A and B) Time course of α-syn aggregation in worms continuously grown on the annotated diet from larval stage L1 (A) or after food switching at the L4 (B). ^∗∗∗∗^p < 0.0001, ^∗∗∗^p = 0.0002. Data shown are mean ± SEM, n = 25 worms per time point per condition. (C and D) Immunoblotting analysis (C) and quantification (D) of α-syn versus β-tubulin levels of protein extracts from day 1 to day 10 adult worms grown on the annotated diet from the L1 (left and middle) or L4 stage (right). Data were normalized to α-syn/β-tubulin levels of day 1 adults worms fed with *E. coli*. (E) Locomotion analysis (thrashing rate) of worms after the food switching at L4 from *E. coli* to *B. subtilis* PXN21. ^∗^p = 0.0152, ^∗∗^p = 0.0072, ^∗∗∗∗^p < 0.0001. Mean values ± SEM, n = 50 worms per condition from two independent experiments are shown (F and G). Time course of α-syn aggregation in worms continuously grown (F) or after the food switching at L4 (G) onto *B. subtilis* strains 168, JH642, NCIB 3610, and PXN21. Black asterisks indicate comparison with *E. coli*; green asterisks denote comparison of PXN21 with NCIB 3610; ^∗∗∗∗^p < 0.0001, ^∗∗∗^p < 0.001, ^∗∗^p < 0.01. Data shown are mean ± SEM, n = 25 worms per time point per condition. (H) Longevity of α-syn worms fed on mixed lawns of the different *B. subtilis* strains shown in (F). ^∗∗∗∗^p < 0.0001, all strains versus *E. coli*. n ≥ 200 worms per condition from three independent experiments. Data shown are mean ± SEM from one representative experiment out of three with similar results, unless stated otherwise.

When *C. elegans* were grown continuously on *E. coli* after hatching, aggregates were observed as early as the second larval (L2) stage (data not shown) and progressively increased in number up to day 3 of adulthood (Figure 2A). In contrast, in animals continuously grown on *B. subtilis* PXN21, there was a near-complete absence of aggregation until day 1 of adulthood, followed by a delayed increase in the number of foci up to day 5 and a subsequent decline. The maximum number of aggregates reached in animals fed with *B. subtilis* was far lower than that observed on the *E. coli* diet, indicating that *B. subtilis* does not simply delay aggregate formation.

In the second feeding condition, when worms were switched from the *E. coli* to the *B. subtilis* diet at the L4 stage, aggregation dropped rapidly, reaching a very low, steady level until day 13 of adulthood (Figure 2B). To test whether the reduction of aggregation had an impact on the fitness of α-syn-expressing animals, we performed locomotion assays in a liquid medium. The locomotion fitness of *C. elegans* was significantly improved after the switch to the *B. subtilis* diet, compared to animals continuously fed on *E. coli*, for a time interval that mirrored the time of reduced aggregation (Figure 2C). We conclude that the most striking effect on aggregation is conferred during continuous growth on *B. subtilis*, with nearly no foci in day 1 of adulthood, whereas the most long-lasting effect is achieved after switching to a *B. subtilis* diet, with aggregation levels remaining low throughout mid- and late adulthood.

The *B. subtilis* diet inhibits aggregation during aging without reducing the expression of α-syn, compared to the *E. coli* diet (Figures 2D and 2E). The decline in aggregation in older *E. coli*-fed worms correlates with the age-dependent decrease in the expression of the *unc-54* promoter (Budovskaya et al., 2008) and, consequently, in α-syn protein levels (Figures 2D and 2E). Remarkably, this decrease is more pronounced in aging worms fed on *E. coli* (Figure 2E), in agreement with previous reports showing a differential diet-dependent regulation of *unc-54* expression (Sánchez-Blanco et al., 2016). Thus, *B. subtilis* inhibits aggregation in aging worms despite the consistently higher levels of α-syn in this diet relative to *E. coli*.

### The Protective Effect against α-Syn Aggregation Is a General Property of *B. subtilis* Species

Previous studies report stress resistance and longevity benefits for wild-type animals grown on various laboratory *B. subtilis* strains (Donato et al., 2017, Garsin et al., 2003, Gusarov et al., 2013, Smolentseva et al., 2017). We therefore asked whether the observed effect on α-syn aggregation is unique to PXN21 or if it is shared among other strains of the *B. subtilis* species. We tested a panel of laboratory *B. subtilis* strains, including 168 (Zeigler et al., 2008), JH642 (Smith et al., 2014), and the undomesticated strain NCIB 3610 (Branda et al., 2001). All strains showed similar effects on α-syn aggregation to the probiotic strain PXN21 following the continuous or food-switching regime (Figures 2F and 2G), indicating that the anti-aggregation effect is a general property of the *B. subtilis* species. Furthermore, all tested *B. subtilis* strains extended the lifespan of α-syn-expressing transgenic animals (Figure 2H; Table S1).

### *B. subtilis* Biofilm Formation and Nitric Oxide Production Protect from α-Syn Aggregation in Aging

*B. subtilis* was previously shown to increase lifespan and stress tolerance in *C. elegans* via several partly co-dependent mechanisms: the formation of a biofilm, a three-dimensional bacterial community embedded in a self-produced extracellular matrix (Branda et al., 2005), in the gut of day 7 adult worms (Donato et al., 2017, Smolentseva et al., 2017); the production of nitric oxide (NO) (Gusarov et al., 2013); and the secretion of colony-stimulating factor (CSF) quorum-sensing pentapeptide (Donato et al., 2017). We first confirmed that the yet-uncharacterized *B. subtilis* strain PXN21 was very proficient at forming a hydrophobic biofilm under standard conditions, similar to the well-characterized *B. subtilis* NCIB 3610 (Figure S2A).

**Figure 3:**
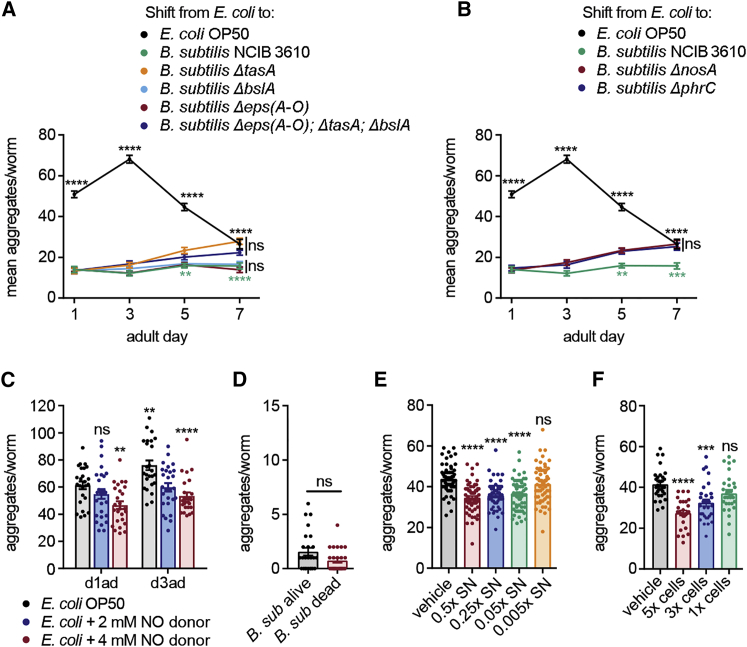
Biofilm Formation and Active Metabolites Contribute to the *B. subtilis* Effect (A) Time course of α-syn aggregation of worms fed with *E. coli* or switched from *E. coli* to *B. subtilis* wild-isolate NCBI3610 and its isogenic-biofilm mutant derivatives: *Δeps(A-O)*, *ΔbslA*, *ΔtasA*, and the triple mutant. Black asterisks show comparisons versus *E. coli*; green asterisks indicate the differences between *B. subtilis* NCIB 3610 and its isogenic mutants; ^∗∗∗∗^p < 0.0001; n ≥ 25 worms per time point per condition. (B) Time course of α-syn aggregation of worms fed with *E. coli* or switched from *E. coli* to *B. subtilis* wild isolate NCBI3610 or its nitric oxide (NO) and quorum-sensing peptide (CSF)-deficient mutants *ΔnosA* and *ΔphrC*, respectively.; ^∗∗∗∗^p < 0.0001, ^∗∗^p = 0.0054/0.0098, ^∗∗∗^p < 0.001; n ≥ 25 worms per time point per condition. (C) Quantification of α-syn aggregates of worms grown from the L1 on *E. coli* supplemented with vehicle (water) or NO donor MAHMA NONOate. ^∗∗∗∗^p < 0.0001, ^∗∗^p < 0. 01; n = 25 worms per time point per condition. (D) Quantification of α-syn aggregates in the head of day 1 adult worms fed with either alive or UV+antibiotic-killed *B. subtilis* PXN21 cells. Unpaired t test; n = 25 worms per condition. (E and F) Quantification of α-syn aggregates of day 1 adult worms grown from the L1 on *E. coli* supplemented with crude extracts from the supernatant (SN) (E) or pelleted cells (cells) (F) of PXN21 cultures (vehicle: ethyl acetate). ^∗∗∗∗^p < 0.0001, ^∗∗∗^p = 0.0001; SN, n = 60 worms per condition from three independent experiments; cells, n = 30 worms per condition from two independent experiments. Data shown are mean ± SEM from one representative experiment out of three with similar results, unless stated otherwise. ns, no significant differences.

To explore whether any of the above bacterial pathways regulating lifespan and stress resistance in *C. elegans* were also responsible for reducing α-syn aggregation in our model, we applied the food-switching approach with *B. subtilis* NCBI 3610 alongside the biofilm-deficient derivatives *Δeps(A-O)*, *ΔbslA*, and *ΔtasA* (Figure S2A). Each of these strains lacks a different extracellular matrix component essential for biofilm formation: *Δeps(A-O)* is defective in exopolysaccharide formation (Branda et al., 2001); *ΔtasA* lacks protein fibers (Romero et al., 2010); and *ΔbslA* is deficient in forming the hydrophobic surface layer that surrounds the biofilm (Hobley et al., 2013, Kobayashi and Iwano, 2012). We found no effect of biofilm mutations on aggregation in the early days of adulthood (Figures 3A and S2B). In contrast, after day 5 of adulthood, when biofilms form in the gut of the nematodes, α-syn aggregation progressively increased when animals were fed the *ΔtasA* strain, but not the *Δeps(A-O)* or *ΔbslA* deletion strains (Figures 3A and S2B). The triple-mutant strain combining all three biofilm deletions (*Δeps(A-O)*, *ΔtasA*, and *ΔbslA*) did not further increase aggregation. We conclude that the biofilm matrix protein TasA supports the ability of *B. subtilis* to protect against α-syn aggregation later in adulthood.

Similar to the *ΔtasA* biofilm-deficient strain, deletion strains for *ΔphrC*, defective in the production of the quorum-sensing pentapeptide CSF, and *Δnos*, defective in NO production, also showed an increase in aggregates later in adulthood but not in earlier stages when compared to the wild-type strain (Figures 3B and S2C). These results are in agreement with previous reports that NO and CSF production is increased by an order of magnitude under biofilm-forming conditions (Donato et al., 2017).

Given that the *ΔphrC* and *Δnos* deletion results implicate CSF and NO in the prolonged protective effect of *B. subtilis* against α-syn aggregation, we asked whether exogenous supplementation of these metabolites in the absence of biofilm could exert a protective effect earlier in adulthood. Whereas animals grown on *E. coli* supplemented with CSF showed no changes in aggregation under the tested conditions (data not shown), NO directly supplied to the worm’s diet induced a significant reduction of aggregation on day 3 of adulthood (Figure 3C). In summary, biofilm-associated bacterial pathways/metabolites responsible for lifespan extension are required for keeping aggregation levels low during aging; however, they do not explain the strong protection observed in early adults.

### A Bacterial Metabolite from *B. subtilis* Inhibits α-Syn Aggregation in Early Adults

Stress resistance and longevity effects induced by *B. subtilis* in *C. elegans* were shown to require live bacteria colonizing the nematode’s gut (Donato et al., 2017, Garsin et al., 2003, Gusarov et al., 2013, Smolentseva et al., 2017). In our case, these mechanisms seem to be relevant only for the effect of *B. subtilis* against α-syn aggregation in late adulthood and cannot explain the strong protection seen in early adulthood, when no biofilm is present and only insufficient levels of NO are likely available from ingested *B. subtilis*. To address whether the effects of *B. subtilis* in early adulthood required live bacteria, we fed α-syn-expressing worms dead *B. subtilis*, killed by a combination of UV and antibiotics. Surprisingly, dead *B. subtilis* were as protective as live bacteria at day 1 of adulthood (Figure 3D).

We next considered whether we could recapitulate the protective effect in the absence of bacteria by supplementing the worms’ diet with *B. subtilis* extracts. Nematodes grown from the L1 on an *E. coli* diet supplemented with *B. subtilis* crude extracts from either the supernatant or pelleted vegetative cells showed a 17%–21% and 21%–33% reduction in aggregation, respectively (Figures 3E and 3F). Therefore, the effect of *B. subtilis* on α-syn aggregation in early adults is partially mediated by the action of an active and stable bacterial metabolite, unlike the short-lived NO, associated with the suppression of aggregation later in life.

### *B. subtilis* Spores and Vegetative Cells Both Protect against α-Syn Aggregation

Bacterial metabolic state is affected by environmental conditions and can strongly influence bacteria-host interactions. *B. subtilis* can exist in two distinct metabolic states: (1) as metabolically active, dividing vegetative cells in nutrient-rich conditions, and (2) as dormant, environmentally resistant spores in nutrient-poor or hostile environments (Nicholson and Setlow, 1990). Under our regular experimental conditions, *B. subtilis* forms lawns that contain a mix of spores and vegetative cells (Figure S3A). Both forms were previously shown to confer longevity and stress-resistance benefits in *C. elegans* via distinct mechanisms (Donato et al., 2017, Gusarov et al., 2013, Sánchez-Blanco et al., 2016, Smolentseva et al., 2017).

**Figure 4:**
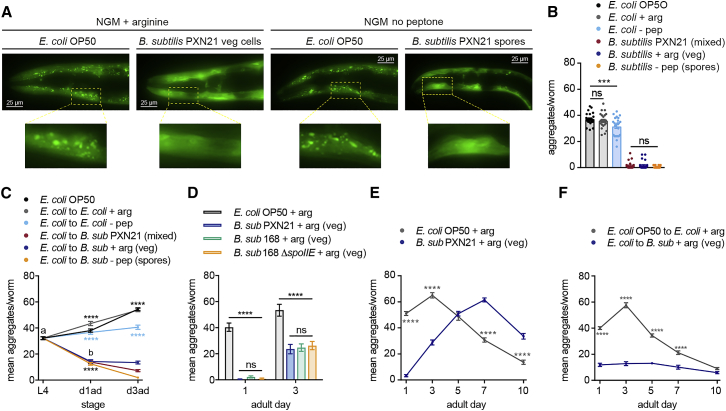
*B. subtilis* Spores and Vegetative Cells Both Protect against α-Syn Aggregation (A) Representative fluorescent images of the head region of day 1 adult worms fed on *E. coli* or *B. subtilis* PXN21 vegetative cells or pure-spore cultures. Higher magnifications of the highlighted regions are shown. NGM, nematode regular growth media; NGM + arginine (+ arg) to inhibit sporulation; NGM no peptone, (- pep) to prevent spore germination. (B) Quantification of α-syn aggregates of day 1 adults worms fed with the different diets. ^∗∗∗^p < 0.001; n = 25 worms per condition. (C) Average number of α-syn aggregates of worms before and after the switching, from *E. coli* to *B. subtilis* lawn of mixed cells, vegetative cells or spores only. ^∗∗∗∗^p < 0.0001 indicates comparison of each diet versus its respective *E. coli* control; a versus b, ^∗∗∗∗^p < 0.0001; n = 25 worms per time point per condition. (D) Average number of α-syn aggregates of worms fed with *E. coli*, *B. subtilis* PXN21, *B. subtilis* 168 strain, or the sporulation mutant 168 *ΔSpoIIE.*^∗∗∗∗^p < 0.0001; n = 25 per time point per condition. (E and F) Time course of α-syn aggregation in worms grown from the L1 (E) or shifted at the L4 stage (F) to *E. coli* or *B. subtilis* vegetative cells. ^∗∗∗∗^p < 0.0001, n = 25 worms per time point per condition. Data shown are mean ± SEM from one representative experiment out of three with similar results, unless stated otherwise. ns, no significant differences.

To determine whether the effect of *B. subtilis* PXN21 on α-syn aggregation depends on the presence of either spores or vegetative cells, we used selective media to acquire pure cultures of each state (see Method Details and Figure S3A). We found that both *B. subtilis* vegetative cells and spores fully prevented aggregation in day 1 adult worms, similar to the mixed lawns (Figures 4A and 4B), and both reversed preformed aggregates (Figures 4C and S3B). The reduced aggregation on the vegetative cell diet was not due to lower α-syn expression (Figures S3C–S3E).

We corroborated the protective effect of *B. subtilis* vegetative cells in early adulthood using the strain 168 carrying a deletion in the *spoIIE* gene, required for sporulation (York et al., 1992). α-syn expressing animals grown on vegetative cells of 168 Δ*spoIIE* strain showed similar levels of aggregation, compared to those grown on vegetative cells of the wild-type *B. subtilis* strain 168 (Figures 4D and S3F).

Finally, we assessed the effect of vegetative cells of *B. subtilis* PXN21 on α-syn aggregation during aging. Worms grown continuously on vegetative cells showed a general delay in the formation of aggregates, but the number of aggregates eventually reached a maximum comparable to that of worms fed on *E. coli* (Figure 4E). In contrast, when worms were shifted at the L4 stage from *E. coli* to a *B. subtilis* lawn of vegetative cells, the aggregation levels remained low until late in adulthood (Figure 4F), similar to those of a mixed lawn diet (Figure 2B).

As both vegetative cells and spores are protective, we next investigated whether they protect through similar or different mechanisms, focusing first on the known lifespan-extending pathways, dietary restriction (DR), and the insulin-like signaling (ILS) pathway.

### Spores Induce DR and Vegetative Cells Protect via a DR-Independent Mechanism

**Figure 5:**
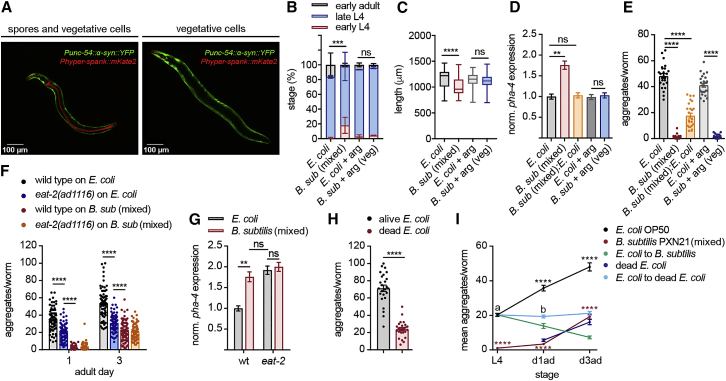
*B. subtilis* Reduces α-Syn Aggregation through Dietary-Restriction-Dependent and Independent Mechanisms (A) Fluorescent images of α-syn worms fed on transgenic NCIB 3610 *B. subtilis* expressing *amyE::Phyper-spank-mKate2*. Spores resistant to digestion can be seen in the entire gut in red (left); vegetative cells are present only before the pharyngeal grinder (right). (B and C) Developmental stage at 48 h (B) and body size at 72 h (C) of α-syn-expressing worms grown on *E. coli* or *B. subtilis* mixed-cell lawns or vegetative cells. ^∗∗∗^p = 0.0007, ^∗∗∗∗^p < 0.0001; n ≥ 80 worms for developmental stage and n ≥ 80 worms for body length per condition from three independent experiments. (D) Normalized *pha-4* expression levels by qRT-PCR in young adult worms grown on the different diet conditions. *pha-4* expression level in worms fed with *E. coli* was taken as 1. ^∗∗^p = 0.0059; n = 3 samples per condition, with three technical replicates each (each sample consisting of ∼4,000 worms). (E) Quantification of α-syn aggregates in day 1 adult worms fed on *E. coli*, *B. subtilis*, or a 1:1 mixture (*B. subtilis*: *E. coli*). ^∗∗∗∗^p < 0.0001; n = 25 worms per condition. (F) Quantification of α-syn aggregates per animal of wild-type or *eat-2(ad456)* worms grown on *E. coli* or *B. subtilis* mixed-cell lawn. ^∗∗∗∗^p < 0.0001; n = 75 worms per time point per condition from three independent experiments. (G) Normalized *pha-4* expression levels by qRT-PCR of young adult wild-type or *eat-2(ad456)* worms grown on the diet conditions shown in (F). ^∗∗^p = 0.0096, n = 3 samples per condition, with three technical replicates each (each sample consisting of ∼4,000 worms). (H) Quantification of α-syn aggregates of day 1 adult worms fed on low concentrations of freshly alive or UV-killed *E. coli*. ^∗∗∗∗^p < 0.0001, n = 25 worms per condition. (I) Average α-syn aggregates of worms before and after L4 switching to *E. coli*, *B. subtilis* mixed lawns, or UV-killed *E. coli* 48 h after seeding. L4, larval stage 4; d1ad, day 1 adult; d3ad, day 3 adult. ^∗∗∗∗^p < 0.0001 comparison versus *E. coli;* a versus b, ns for *E. coli* to UV-killed *E. coli* versus *E. coli* versus, ^∗∗∗∗^p < 0.0001 for *E. coli* to *B. subtilis* versus *E. coli*; n = 25 worms per time point per condition. Data shown are mean ± SEM from one representative experiment out of three with similar results, unless stated otherwise. ns, no significant differences.

We first considered that DR may underlie the specific protective effects of spores against aggregation, as *C. elegans* is virtually unable to digest spores (Laaberki and Dworkin, 2008) (Figures 5A and S4A). A *B. subtilis* spores-only diet poorly sustained growth, inducing severe signs of DR, which is reflected by a strong delay in development to adulthood by 5–7 days and a significantly smaller size in adult worms, compared to those grown on *E. coli* (Figure S4B). Worms grown on mixed *B. subtilis* lawns showed mild DR, manifested by a slight developmental delay and smaller body size compared to those grown on *E. coli* (Figures 5A–5C, S4B, and S4C). In contrast, worms fed only with vegetative cells did not show any signs of DR (Figures 5A–5C, S4B, and S4C). In addition, only small brood size differences between the two diets were observed, which disappeared in the food-switching condition (Figures S4D–S4G). This rules out diet effects on fecundity as a contributing factor to the reduction of aggregation.

We confirmed that *B. subtilis* mixed lawns induced a state of DR using the marker *pha-4*, an ortholog of the FoxA transcription factors (Panowski et al., 2007): there was a significant increase in *pha-4* levels in animals fed on mixed *B. subtilis* lawns, but not on vegetative cells, compared to animals grown on *E. coli* (Figure 5D). Furthermore, when we supplemented *B. subtilis* mixed lawns with *E. coli* at a 1:1 ratio, we saw a strong protection against α-syn aggregation (Figure 5E) in the absence of *pha-4* upregulation (Figure 5D). These results suggest that DR is not responsible for the anti-aggregation effect of vegetative cells, but it may have an effect when animals are fed on spore-rich lawns.

DR was previously shown to suppress proteotoxicity in animal models of polyglutamine and amyloid beta aggregation (Steinkraus et al., 2008), to modify adverse effects of α-syn on the autonomic nervous system in mice (Griffioen et al., 2013), and to alleviate α-syn toxicity in yeast (Guedes et al., 2017). However, to our knowledge, no direct evidence exists that DR can inhibit α-syn aggregation in animal models. We therefore tested whether loss of function of the nicotinic acetylcholine receptor subunit *eat-2*, a genetic mimetic of dietary restriction due to reduced food uptake (Lakowski and Hekimi, 1998, McKay et al., 2004), was able to suppress α-syn aggregate formation. Indeed, *eat-2(ad456)* animals grown on *E. coli* showed less aggregation in day 1 and day 3 adults, compared to wild-type animals grown on *E. coli* (Figure 5F). However, this reduction was much weaker than the one seen in worms grown on *B. subtilis* mixed lawns. In addition, a *B. subtilis* diet further decreased the number of aggregates of *eat-2* mutants (Figure 5F), without further increasing *pha-4* expression levels (Figure 5G). Similar effects were obtained with a *B. subtilis* vegetative cell diet (Figures S4H and S4I).

We further confirmed that DR is able to inhibit α-syn aggregation by feeding worms with limited amounts of *E. coli* killed by UV, a known experimental way to induce DR in *C. elegans* (Greer et al., 2007) (Figures 5H, S4J, and S4K). However, shifting worms fed *ad libitum* on *E. coli* until the L4 stage (or until day 1 of adulthood) to a DR-inducing UV-killed *E. coli* condition did not clear preformed aggregates (Figures 5I and S4L), even though it inhibited the formation of new aggregates like the probiotic diet (Figure 5I). Therefore, DR per se does not fully reproduce the sum of *B. subtilis* effects, which include both inhibition and the clearance of aggregates.

Together, these results reveal that DR has a protective role against α-syn aggregation, and it may underlie part of the protective effect triggered by *B. subtilis* spores. However, vegetative cells inhibit and dissolve α-syn aggregates through a DR-independent mechanism.

### DAF-16 Contributes to the Protection of *B. subtilis* Later in Adulthood

**Figure 6:**
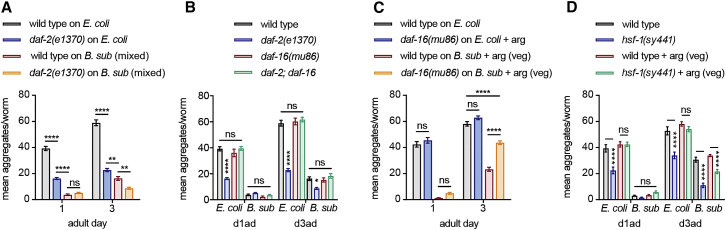
DAF-16 Contributes to the Protection of *B. subtilis* in Aging (A) Quantification of α-syn aggregates in the head of wild-type or *daf-2(e1370)* worms grown on *E. coli* or *B. subtilis* PXN21 mixed-cell lawn. ^∗∗∗∗^p < 0.0001, ^∗∗∗^p < 0.001, ^∗∗^p < 0. 01; n ≥ 25 per time point per condition. (B) Average α-syn aggregates in wild-type, *daf-2(e1370)*, *daf-16(mu86)*, and *daf-2;daf-16(mu86)* double-mutant worms grown on *E. coli* or *B. subtilis* PXN21 mixed-cell lawn (spore-rich). ^∗∗∗∗^p < 0.0001, ^∗^p = 0.0358; n ≥ 25 per time point per condition. (C) Average α-syn aggregates in wild-type and *daf-2;daf-16(mu86)* worms grown on *E. coli* or vegetative *B. subtilis* PXN21 lawn. ^∗∗∗∗^p < 0.0001; n = 25 per time point per condition. (D) Average α-syn aggregates in wild-type and *hsf-1(sy441)* mutant worms grown on *E. coli* or mixed-cell *B. subtilis* PXN21 lawns or vegetative-only diet (+ arg). ^∗∗∗∗^p < 0.0001; n = 25 per time point per condition. Data shown are mean ± SEM from one representative experiment out of three with similar results, unless stated otherwise. ns, no significant differences.

A *C. elegans* lifespan extension by *B. subtilis* was previously linked to the downregulation of the evolutionarily conserved ILS pathway (Donato et al., 2017). Decreased signaling of the insulin growth factor (IGF) receptor DAF-2 (Kenyon et al., 1993, Kimura et al., 1997) extends lifespan by activating two downstream transcription factors, DAF-16/FOXO (Lin et al., 1997, Ogg et al., 1997) and HSF-1 (Hsu et al., 2003). A reduced ILS also protects worms from stress conditions such as toxic protein aggregation of polyglutamine stretches (Hsu et al., 2003, Morley et al., 2002), amyloid beta (Cohen et al., 2006), and α-syn (Knight et al., 2014). To determine whether the ILS pathway plays a role in the *B. subtilis-*triggered protection against α-syn aggregation, we used *daf-2(e1370)* mutant worms with inhibited ILS signaling (Gems et al., 1998). The *daf-2(e1370)* α-syn-expressing animals grown on *E. coli* showed a strong suppression of aggregates in day 1 and day 3 adults compared to wild-type animals (Figure 6A), confirming previous reports (Knight et al., 2014). However, the *daf-2* protective effect was significantly less pronounced than that seen in *B. subtilis* PXN21-fed wild-type worms, and the *B. subtilis* diet further reduced aggregation levels of *daf-2(e1370)* animals (Figure 6A). The additive effect between the *B. subtilis* diet and *daf-2* downregulation indicates that *B. subtilis* acts through an ILS-independent pathway.

To further investigate the role of the ILS pathway, we analyzed the role of DAF-16/FOXO transcription factor and found that the *daf-16(mu86)* loss-of-function mutation (Lin et al., 1997) fully abrogated the *daf-2(e1370)* protective effect on *E. coli* (Figure 6B). In contrast, *daf-16(mu86)* did not affect the efficiency of a *B. subtilis* mixed diet to inhibit aggregation (Figure 6B). Similarly, no increase in aggregation levels was observed in day 1 adults in *daf-16* mutant worms fed with *B. subtilis* vegetative cells (Figure 6C). However, in day 3 adult worms fed on vegetative cells, loss of DAF-16 function led to a faster increase in the number of aggregates (Figure 6C), indicating that the later protection triggered by the vegetative cell diet relies partially on the activity of DAF-16. The *hsf-1(sy441)* mutation, which inhibits the second major transcription factor downstream of DAF-2, did not increase aggregation levels when grown on any *B. subtilis* diet (Figure 6D).

In conclusion, the effect of *B. subtilis* on α-syn aggregation is independent of the ILS pathway in early adults. However, the protective effect later in adulthood induced by vegetative *B. subtilis* cells is mediated in part by the action of DAF-16. Thus, our results further indicate that *B. subtilis* spores and vegetative cells act redundantly through distinct protective mechanisms, with spores acting likely via PHA-4/DR and vegetative cells via DAF-16.

### *B. subtilis* Inhibits α-Syn Aggregation by Altering Sphingolipid Metabolism in the Host

**Figure 7:**
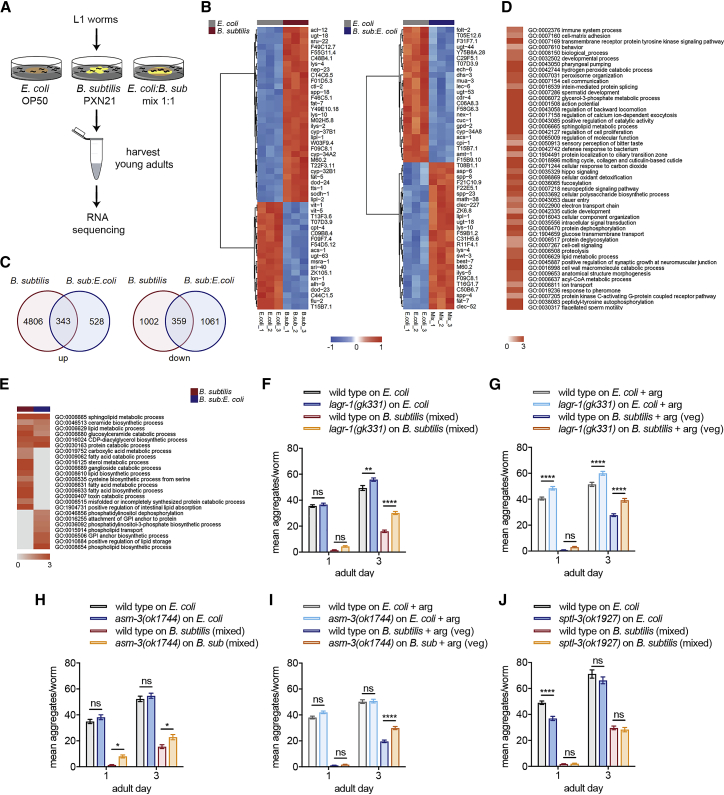
*B. subtilis* Protects against α-Syn Aggregation by Changing the Sphingolipid Metabolism in the Host (A) Assay strategy for the comparative transcriptomics experiment. (B) Heatmap showing the top 50 most differentially expressed genes by false discovery rate (FDR) between *E. coli* and *B. subtilis* PXN21 or *E. coli* and *B. subtilis:E. coli* mix. A fold change ≥ 1.5, p < 0.05, and FDR < 0.05 were considered for statistical significance. (C) Venn diagrams showing the overlap between the statistically significant upregulated and downregulated genes in *B. subtilis* PXN21 versus the mix of *B. subtilis* and *E. coli* diet. (D) Summary of the top 50 statistically significant non-redundant BP GO terms of *B. subtilis* PXN21 versus *E. coli* by log_10_ p value. (E) Lipid-metabolism-related BP GO terms upregulated by *B. subtilis* PXN21 and the mix versus *E. coli* diets by log_10_ p value. Commonly upregulated lipid GO terms (top), *B. subtilis* exclusive (middle), and exclusive for the mix of *B. subtilis* and *E. coli* diet (bottom) are shown. Gray indicates processes not differentially regulated. (F–J) Average α-syn aggregates of wild-type or mutant animals for sphingolipid metabolism genes: *lagr-1(gk331)* fed from the L1 with *B. subtilis* PXN21 mixed lawn diet (F) or vegetative cells (G); *asm-3(ok1744)* mutant animals fed from the L1 with *B. subtilis* PXN21 mixed lawn diet (H) or vegetative cells (I); *sptl-3(ok1927)* mutant animals fed from the L1 with *B. subtilis* PXN21 mixed lawn diet (J), compared to *E. coli*. ^∗∗∗∗^p < 0.0001, ^∗∗^p < 0.01, ^∗^p < 0.01; ns, no significant differences. Mean values ± SEM, n = 50 worms per time point per condition from two independent experiments are shown.

To uncover the host response pathways that are modified by *B. subtilis* to induce the protective effect, we performed comparative global transcriptomics analysis (RNA sequencing [RNA-seq]) to compare young adult animals fed on two different diets: *E. coli* OP50 and *B. subtilis* PXN21 mixed state (Figure 7A). In addition, since the mixture of *E. coli* and *B. subtilis* retained much of the anti-aggregation effect but did not induce DR (see Figure 5E), we included this condition in the transcriptomics experiment to reveal DR-independent protective mechanisms.

We found that 6,510 genes were differentially expressed by 1.5-fold change or higher in animals fed with *B. subtilis* compared to those fed *E. coli* (false discovery rate [FDR] < 0.05, p value < 0.05) (Table S2). A summary of the top 50 most differentially expressed genes between *B. subtilis-* and *E. coli*-fed animals, ranked by lowest FDR, is shown in Figure 7B. Sample clustering showed that the mix of both bacteria exhibited a gene expression profile closer to that of animals fed on *E. coli* than on *B. subtilis* (Figure S5A). In agreement with this, only 2,291 genes were found to be differentially expressed in this case (Figure 7C; Table S3). Of these, 343 genes were commonly upregulated and 359 downregulated in both animals fed with *B. subtilis* and the mixture of the two bacteria, compared to *E. coli* (Figure 7C; Tables S4 and S5). The RNaseq results were validated by randomly selecting 10 upregulated and downregulated genes and testing the level of expression by qRT-PCR (Figures S5B and S5C). As expected, *pha-4* was significantly upregulated (1.35-fold change) only in animals fed on *B. subtilis*, compared with those fed on *E. coli* (Figure S5D), but showed no differences in animals fed on the mix of the two bacteria versus *E. coli*. No other DR-related transcription factors were differentially regulated at the transcript level in the different diets (Figure S5D).

Previous genome-wide screens on *C. elegans* models have identified modifiers of α-syn aggregation (Hamamichi et al., 2008, Knight et al., 2014, van Ham et al., 2008). We intersected our transcriptomics datasets with these lists and found that a number of known suppressors of aggregation were upregulated by the *B. subtilis* diet (Table S6), indicating that *B. subtilis* may impart its effects on α-syn through the activation of multiple protective pathways.

Next, we performed a Gene Ontology (GO) term analysis of gene sets affected by the two bacterial diets and found that 708 and 506 biological process (BP) terms were differentially regulated by *B. subtilis* and by the mix of the two bacteria, respectively (Table S7). The summaries of the 50 most significant non-redundant upregulated GO terms in *B. subtilis* and the mix versus *E. coli*, processed by REduce & VIsualize Gene Ontology (REVIGO), are shown (Figures 7D and S5E). Among the top upregulated biological pathways by *B. subtilis* PXN21 are immune system processes, protein localization, redox processes, general metabolism, and, in particular, lipid metabolism. An expanded analysis of lipid-related terms revealed that several lipid-metabolism-related processes are significantly upregulated in both *B. subtilis* and the mixed diet, compared to *E. coli* (Figure 7E).

We focused on a specific pathway branch of lipid metabolism, the sphingolipid metabolism pathway, as it has been proposed to modify α-syn pathology in PD (Alecu and Bennett, 2019, Galvagnion, 2017, Lin et al., 2019, Plotegher et al., 2019). Ceramide lipid metabolism is the central hub of the sphingolipid metabolic pathway and was upregulated by both *B. subtilis* and mixed diets, with a p value <0.001 (Figures 7E and S5F). Genes in this pathway that are upregulated by *B. subtilis* (Figure S5F; Table S8) include *lagr-1*, a *C. elegans* ortholog of human ceramide synthase CERS1 (Deng et al., 2008, Jiang et al., 1998), and *asm-3* (Kim and Sun, 2012), an ortholog of human acid sphingomyelinase, SMPD1, which hydrolyses sphingomyelin to ceramide. Among the downregulated genes, we identified *sptl-3,* an ortholog of human SPTLC2, a serine palmitoyltransferase that catalyzes the first and rate-limiting step of the ceramide *de novo* biosynthesis pathway (Miyake et al., 1995).

To address the functional significance of the altered expression of ceramide pathway genes by *B. subtilis*, we used loss-of-function mutations of *lagr-1*, *asm-3*, and *sptl-3.* Loss of the upregulated genes *lagr-1* or *asm-3* increased the number of aggregates in worms continuously grown on *B. subtilis* (Figures 7F–7I). Conversely, disruption of *sptl-3*, which was downregulated by *B. subtilis*, reduced aggregation on the *E. coli* diet compared to wild-type worms (Figure 7J).

Sphingolipid metabolism genes were previously reported to be regulated downstream of *eat-2-*induced DR (Calvert et al., 2016). Our data indicate that several of the sphingolipid metabolism genes are regulated also in the *B. subtilis* feeding condition that does not induce DR. Thus, we conclude that both DR-dependent and DR-independent effects of the *B. subtilis* diet converge on sphingolipid metabolism. In light of our findings, we propose that alterations in sphingolipid metabolism triggered by the *B. subtilis* diet result in a reduction of α-syn aggregation in *C. elegans*.

## Discussion

The accumulation of misfolded α-syn into pathological aggregates plays a central role in the pathogenesis of PD and other synucleinopathies (Alafuzoff and Hartikainen, 2017). Significant effort has been invested into finding ways to suppress the formation or enhance the clearance of toxic α-syn aggregates as a treatment for PD (Savitt and Jankovic, 2019), though no such therapies are available yet. Previous studies suggest that the presence of distinct groups of bacteria in the gut microbiome modulate PD pathology (Minato et al., 2017, Sampson et al., 2016, Scheperjans et al., 2015). However, deciphering the precise effect of individual bacterial species remains challenging. In this study, we show that *B. subtilis* PXN21, a probiotic strain that is available for human consumption, both inhibits aggregation and efficiently removes preformed aggregates in a *C. elegans* model with ectopic expression of human α-syn.

It was previously reported that biofilm formation and NO production by *B. subtilis* confers *C. elegans* with stress resistance and enhanced longevity (Donato et al., 2017, Smolentseva et al., 2017). Our results reveal that while these pathways contribute to the suppression of α-syn later in life, the protective effect seen earlier in life is independent of these mechanisms. In young adults, the probiotic acts independently of gut colonization and triggers its protective effects partly via the production of bacterial metabolites other than NO.

We provide evidence that distinct metabolic states of the bacteria affect the physiology of the host as well as α-syn aggregation in different ways. *B. subtilis* spores, which are resistant to digestion and are metabolically inert, induce DR. DR conditions are known to activate the lysosomal autophagy pathway (Levine and Kroemer, 2008), one of the main systems of α-syn clearance in cells (Poewe et al., 2017). We find that DR is an effective mechanism to inhibit the accumulation of α-syn in *C. elegans* and is therefore a likely partial mechanism of action of *B. subtilis* spores. In contrast, *B. subtilis* vegetative cells protect via a DR-independent mechanism that partly depends on the action of DAF-16 in older animals. Downregulation of the ILS pathway, although implicated in the lifespan-extending effects of *B. subtilis*, is not required for the early protection against α-syn. Therefore, the anti-aggregation properties of *B. subtilis* remain, to a large extent, distinct from its anti-aging effects.

Our transcriptomics analysis revealed that part of the probiotic’s effect is mediated by alterations in the sphingolipid metabolism pathway, particularly the regulation of the enzymes LAGR-1/CERS1 (ceramide synthase), ASM-3/SMPD1 (acid sphingomyelinase), and SPTL-3/SPTLC2 (serine palmitoyltransferase). Previous studies suggest that an imbalance of lipids, including ceramides and sphingolipid intermediates, may contribute to the pathology of PD. For example, reduced levels of ceramides occur selectively in brain regions affected by PD pathology (Abbott et al., 2014). Several genetic risk loci in PD affect ceramide metabolism and cellular sphingolipid content (Ferrazza et al., 2016, Gan-Or et al., 2013, Henry et al., 2015, Lin et al., 2019, Plotegher et al., 2019), including mutations in ASM-3/SMPD1 (Foo et al., 2013, Gan-Or et al., 2015, Gan-Or et al., 2013, Ylönen et al., 2017) and GBA (the lysosomal glucocerebrosidase). Furthermore, ASM-3/SMPD1 deficiency in cell-based models was shown to lead to α-syn accumulation (Alcalay et al., 2019), whereas inhibition of the *Drosophila melanogaster* ortholog of SPTL-3/SPTLC2 was found to suppress α-syn-associated neurodegenerative phenotypes (Lin et al., 2018). Furthermore, direct interactions between α-syn and lipids are known to modulate the aggregation propensity of this protein both *in vitro* and *in vivo* (Galvagnion, 2017). We propose that the *B. subtilis* probiotic diet in the *C. elegans* model alters the lipid composition of the cell, directly affecting α-syn aggregation. Our data further demonstrate that a simple dietary intervention can concurrently affect several branches of the sphingolipid pathway, to beneficial effect.

PD is typified by the presence of intraneuronal α-syn aggregation and dopaminergic degeneration (Poewe et al., 2017). Our current study is based on an established *C. elegans* model that expresses human α-syn in muscle cells, which allows us to assess aggregation *in vivo*. The effects of *B. subtilis* on the nervous system, as well as its efficacy in mouse models of PD, present promising avenues of future investigation. The prospect of *B. subtilis* modifying α-syn aggregation in humans could open exciting possibilities for diet-based, disease-modifying interventions through the manipulation of microbiome composition in the gastrointestinal tract or the development of drug therapies based on protective bacterial metabolites.

## STAR★Methods

### Key Resources Table

**Table:** 

REAGENT or RESOURCE	SOURCE	IDENTIFIER
**Antibodies**		

Mouse monoclonal antibody anti-α-synuclein	BD Biosciences	Cat# 610786; RRID: AB_398107
Mouse monoclonal anti-β-actin	Sigma-Aldrich	Cat# A5441; RRID: AB_476744
Mouse monoclonal anti-β-Tubulin	Sigma-Aldrich	Cat# T4026; RRID: AB_477577
Rabbit Polyclonal Anti-Mouse	Agilent	Cat# P0260; RRID: AB_2636929

**Bacterial and Virus Strains**		

*E. coli*: OP50	CGC	RRID:WB-STRAIN:OP50
*B. subtilis*: probiotic PXN21	Bio-Kult by ADM-Protexin	N/A
*B. subtilis*: NCIB 3610 Marburg, undomesticated	BGSC	BGSCID: 3A1
*B. subtilis*: NRS2097 NCIB 3610 *ΔbslA::cmlR*	Nicola Stanley-Wall (Ostrowski et al.,2011)	N/A
*B. subtilis*: NRS2415 NCIB 3610 *ΔtasA::spcR*	Nicola Stanley-Wall (Ostrowski et al.,2011)	N/A
*B. subtilis*: NRS2450 NCIB 3610 *Δeps(A-O)::tetR*	Nicola Stanley-Wall (Ostrowski et al.,2011)	N/A
*B. subtilis*: NRS2543 NCIB 3610 *Δeps(A-O)::tetR ΔtasA::spcR ΔbslA::cmlR*	Nicola Stanley-Wall (Ostrowski et al.,2011)	N/A
*B. subtilis*: NCIB 3610 *amyE Phyper-spank-mKate2::spcR*	Ákos T. Kovács (van Gestel et al., 2014)	N/A
*B. subtilis:* NRS5852 NCIB 3610 *amyE::Phyper-spank-mKate2::spcR*	Nicola Stanley-Wall	N/A
*B. subtilis*: NRS6296 NCIB 3610 *ΔnosA::kanR*	Nicola Stanley-Wall	N/A
*B. subtilis*: NRS6297 NCIB 3610, *ΔphrC::kanR*	Nicola Stanley-Wall	N/A
*B. subtilis*:168 *trpC2; ΔspoIIE::kanR*	Addgene-BGSC (Koo et al.,2017)	Cat# 1000000115
		BGSCID: BKK00640
*B. subtilis*:168 *trpC2; ΔnosA::kanR*	Addgene-BGSC (Koo et al.,2017)	BGSCID: BKK07630
*B. subtilis*: JH642	BGSC	BGSCID: 1A96
Bacteriophage*: Bacillus* phage SPP1	Anne Moir (University of Sheffield)	N/A

**Chemicals, Peptides, and Recombinant Proteins**		

Agar	Formedium	Cat# AGA02
Select Agar	Thermo Fisher Scientific	Cat#30391023
Bacto peptone	BD Biosciences	Cat# 211677
Sodium Hypochlorite solution (4.00-4.99%)	Honeywell	Cat# 239305
Ca(NO_3_)_2_	Sigma-Aldrich	Cat# C1396
CaCl_2_	Sigma-Aldrich	Cat# 449709
Coomassie Brilliant Blue G-250	Thermo Fisher Scientific	Cat# 20279
Schaeffer and Fulton Spore Stain Solution A	Sigma-Aldrich	Cat# 90903
Schaeffer and Fulton Spore Stain Solution B	Sigma-Aldrich	Cat# 39955
Dichloromethane (DCM)	Thermo Fisher Scientific	Cat# 402152
Nutrient broth No 3	Sigma-Aldrich	Cat# 70149
Dried Milk Powder	Marvel	N/A
Dithiothreitol (DTT)	GE Healthcare	Cat# 17-1318-01
Ethanol	ACROS Organics	Cat# AC615095000
Ethyl acetate	Sigma-Aldrich	Cat# 319902
EDTA	Sigma-Aldrich	Cat# 798681
FeCl_3_	Sigma-Aldrich	Cat# 451649
FeSO_4_	Sigma-Aldrich	Cat# 450278
L-glutamic acid monosodium salt monohydrate	Sigma-Aldrich	Cat# 49621
Glycerol ≥ 99.5%	Thermo Fisher Scientific	Cat# BP229-1
HEPES sodium salt	Sigma-Aldrich	Cat# H7006
Hydrogen peroxide solution	Sigma-Aldrich	Cat# 216763
Kanamycin Sulfate	Corning	Cat# 61-176-RG
KCl	Sigma-Aldrich	Cat# P9541
KH_2_PO_4_	ACROS Organics	Cat# AC424200025
K_2_HPO_4_	ACROS Organics	Cat# AC424190025
L-Arginine	Alfa Aesar	Cat# A15738
Levamisole Hydrochloride	MP Biomedicals	Cat# 155228
Luminol 97%	Sigma-Aldrich	Cat# 123072
Luria-Bertani (LB) broth	Sigma-Aldrich	Cat# L3022
Lysozyme	Thermo Fisher Scientific	Cat# 89833
MgCl_2_	Sigma-Aldrich	Cat# M8266
MnCl_2_	Sigma-Aldrich	Cat# 244589
MOPS	Sigma-Aldrich	Cat# M1254
Na_2_HPO_4_	ACROS Organics	Cat#AC204851000
NaCl	Thermo Fisher Scientific	Cat# BP358-1
NaF	Sigma-Aldrich	Cat# S7920
NaOH	Thermo Fisher Scientific	Cat# S612-3
NativePAGE 4-16% Bis-Tris-gels	Thermo Fisher Scientific	Cat# BN1002BOX
NativePAGE 20x Running buffer	Thermo Fisher Scientific	Cat# BN2001
Nitrocellulose membrane 0.2 μm	Biorad	Cat# 1620112
MAHMA NONOate (NO donor)	Sigma-Aldrich	Cat# M1555
NuPAGE 4-12% Bis-Tris-gels	Invitrogen	Cat# NP0322PK2
PageRuler Protein Ladder, 10 to 250 kDa	Thermo Fisher Scientific	Cat# 26620
PBS	Sigma-Aldrich	Cat# P4417
P-Coumaric acid	Sigma-Aldrich	Cat# C9008
Penicillin Streptomycin	GIBCO	Cat# 15070063
PFA	Sigma-Aldrich	Cat#
Protease Inhibitor Mix	GE Healthcare	Cat# 80-6501-23
Proteinase K	BioVision	Cat# 9211-5
Thiamine hydrochloride	Sigma-Aldrich	Cat# T4625
Triton X-100	MP Biomedicals	Cat# 807423
TWEEN® 20	Sigma-Aldrich	Cat# P1379
ZnCl_2_	Sigma-Aldrich	Cat# 229997

**Critical Commercial Assays**		

OneTaq® 2X Master Mix with Standard Buffer	New England Biolabs (NEB)	Cat# M0482S
GoTaq® G2 Green Master Mix	Promega	Cat#M782A
QIAquick PCR Purification Kit	QIAGEN	Cat#28104
Qubit RNA BR kit	Thermo Fisher Scientific	Cat# Q10210
Quick Start Bradford Protein Assay Kit	Bio-Rad	Cat# 5000201
Quick-RNA Microprep Kit	ZYMO Research	Cat# R1050
Q5 High-Fidelity 2X Master Mix	NEB	Cat# M0492S
SuperScript IV First-Strand Synthesis System	Thermo Fisher Scientific	Cat# 18091050
TruSeq Stranded mRNA kit	Illumina	Cat# 20020594
LightCycler® 480 SYBR Green I master	Roche	Cat# 04707516001
Zymoclean Gel DNA Recovery Kit	ZYMO Research	Cat# D4001

**Deposited Data**		

*C. elegans* RNA-Seq reads (fastq files)	This study	ArrayExpress: E-MTAB-8164

**Experimental Models: Organisms/Strains**		

*C. elegans*: N2 Bristol	CGC	CGCRRID:WB-STRAIN:N2
*C. elegans*: NL5901 pkIs2386[P*unc-54*::α-synuclein::YFP + unc-119(+)]	Ellen Nollen (van Ham et al., 2008)	RRID:WB-STRAIN:NL5901
*C. elegans*: MDH586 *daf-2(e1370)* III; pkIs2386	This study	N/A
*C. elegans*: MDH585 *daf-16(mu86)* I; pkIs2386	This study	N/A
*C. elegans*: MDH587 *hsf-1(sy441)* I; pkIs2386	This study	N/A
*C. elegans*: MDH657 *daf-2(e1370)* III; *daf-16(mu86)* I; pkIs2386	This study	N/A
*C. elegans*: MDH611 *eat-2(ad465)* II; pkIs2386	This study	N/A
*C. elegans*: MDH711 *lagr-1(gk331)* I, pkIs2386	This study	N/A
*C. elegans*: MDH724 *asm-3*(ok1744) IV; pkIs2386	This study	N/A
*C. elegans*: MDH725 *sptl-3*(ok1927) II; pkIs2386	This study	N/A

**Oligonucleotides**		

For information regarding oligonucleotide sequences used in this study please refer to Table S9	This study	Table S9

**Software and Algorithms**		

Cutadapt version cutadapt-1.9.dev2	Martin, 2011	https://cutadapt.readthedocs.io/en/stable/
EdgeR package version 3.16.5	Lun et al., 2016, Robinson et al., 2010	https://bioconductor.org/packages/release/bioc/html/edgeR.html
FeatureCounts package version 1.5.3	Liao et al., 2014	http://subread.sourceforge.net
Fiji	Schindelin et al., 2012	http://fiji.sc/
GraphPad Prism	GraphPad Software, La Jolla California USA	https://www.graphpad.com
ImageJ	Schneider et al., 2012	https://imagej.nih.gov/ij/
Limma package version 3.30.13 of Bioconductor	Ritchie et al., 2015	https://bioconductor.org/packages/release/bioc/html/limma.html
Multiple Experiment Viewer version 4.9.0_r2731	Howe et al., 2010	http://mev.tm4.org/
OASIS 2	Han et al., 2016; Oncotarget 11269	https://sbi.postech.ac.kr/oasis2/
Pathview package version 1.24.0 of Bioconductor.	Luo and Brouwer, 2013	https://bioconductor.org/packages/release/bioc/html/pathview.html
Pheatmap package (R package version 1.0.8.)	Kolde, 2015	https://rdrr.io/cran/pheatmap/
Photoshop CC	Adobe Systems Inc.	https://www.adobe.com/Photoshop
Primer3Plus	Untergasser et al., 2007	http://bioinfo.ut.ee/primer3/
R-3.5.1	R Core Team	https://www.r-project.org/
SnapGene	SnapGene software from GSL Biotech	https://www.snapgene.com/
STAR version 2.5.2b	Dobin et al., 2013	http://code.google.com/p/rna-star/
Illustrator CC	Adobe Systems Inc.	https://www.adobe.com/products/illustrator
WormLab tracking platform	MBF Bioscience, Williston, VT USA	https://www.mbfbioscience.com/wormlab

### Lead contact and materials availability

Further information and requests for resources and reagents should be directed to and will be fulfilled by the Lead Contact, Maria Doitsidou (maria.doitsidou@ed.ac.uk).

### Experimental model and subject details

#### Nematode and bacterial strains

All bacterial and nematode strains used and generated in this study can be found in the Key Resources Table.

*C. elegans* NL5901 pkIs2386[P*unc-54*::α-synuclein::YFP + unc-119(+)] was kindly provided by Ellen Nollen. *C. elegans* N2 and all the mutant strains used for the generation of the different pkIs2386 derived strains were obtained from the Caenorhabditis Genetics Center (CGC) (https://cgc.umn.edu) and the Million Mutant collection (Thompson et al., 2013).

Strains obtained from the CGC are: CB1370 *daf-2(e1370)*, CF1038 *daf-16(mu86)*, PS3551 *hsf-1(sy441)*, DA465 *eat-2(ad465)*, VC747 *lagr-1(gk331)*, RB1579 *sptl-3(ok1927)*, RB1487 a*sm-3(ok1744)*.

The molecular identity of these alleles is as follows: *daf-2(e1370)* (Gems et al., 1998) is a missense reference allele, *daf-16(mu86)* (Lin et al., 1997) and *lagr-1(gk331)* (Deng et al., 2008) are deletion, loss of function alleles. *eat-2(ad456)* (Lakowski and Hekimi, 1998) and *hsf1(sy441)* (Hajdu-Cronin et al., 2004) are nonsense alleles. *asm-3(ok1744)* is a complex substitution allele removing most of the last 7 exons, *sptl-3(ok1927)* is a deletion removing exons 5 to 9.

The following strains were generated in this study: MDH586 daf-2(e1370) III; pkIs2386, MDH585 daf-16(mu86) I; pkIs2386, MDH587 hsf-1(sy441) I; pkIs2386, MDH657 daf-2(e1370) III; daf-16(mu86) I; pkIs2386, MDH614 daf-2(gk390525) III; pkIs2386, MDH611 eat-2(ad465) II; pkIs2386, MDH711 lagr-1(gk331) I, pkIs2386, MDH725 sptl-3(ok1927) II; pkIs2386, MDH724 asm-3(ok1744) IV; pkIs2386.

Several bacterial strains were used in this study, *E. coli* OP50 was obtained from the CGC. The *B. subtilis* PXN21 strain (Colenutt and Cutting, 2014) was isolated from Bio-Kult Advanced Multi-Strain Formulation dietary supplement (https://www.bio-kult.com, ADM-Protexin) and genotyped using universal 16S rRNA primers (Lane, 1991) (See Table S9 for primers). The wild-type undomesticated *B. subtilis* NCIB 3610 and the laboratory 168 and JH64102 strains were obtained from the Bacillus Genetic Stock Center (BGSC) (http://www.bgsc.org). 168-based deletion strain *ΔspoIIE* was obtained from Addgene (www.addgene.org) as part of the *B. subtilis* Single Gene Deletion Library with Kanamycin resistance (Koo et al., 2017). The NCIB 3610 deficient derivatives strains *ΔtasA::cml, ΔbslA::spc, Δeps(A-O)::tet*, *Δnos::kan* and *ΔphrC::kan* and the triple *ΔbslA::spc; eps(A-O)::tet; tasA::::cml*, were obtained from the Nicola Stanley-Wall lab. SPP1 phage transductions were used to introduce DNA into *B. subtilis* NCIB 3610 strains from 168 derivatives (Verhamme et al., 2007). Drug resistance cassettes are indicated as follows: cml, chloramphenicol resistance; kan, kanamycin resistance; erm, erythromycin resistance; tet, tetracycline resistance and spc, spectinomycin resistance.

### Method details

#### *C. elegans* growth conditions

Nematodes were handled according to standard practices (Brenner, 1974, Stiernagle, 2006). Worm strains were grown on NGM plates for experiments with mixed spores and vegetative cells, NGM plus 0.5 mM of arginine for experiments with vegetative cells (to avoid sporulation), or NGM without peptone for experiments with spores only (to avoid germination). All strains were grown at 20°C unless otherwise indicated. Worms were synchronized by the alkaline hypochlorite method (Stiernagle, 2006) and left nutating overnight to hatch in M9 supplemented with kanamycin 50 μg/ mL (Sigma) and 1x antibiotic-antimycotic (Thermo Fisher Scientific). For the continuous feeding regime, synchronized L1 worms were plated, grown until day 1 adults, and then transferred to new plates every two days. For the food switch experiments, worms were grown on *E. coli* OP50 until L4 stage, then shifted to a new diet and transferred to new plates every two days thereafter.

#### Bacterial growth conditions

Bacterial cultures were grown until an OD_600_ of 1 in Luria-Bertani (LB) media at 37°C with agitation (220 rpm). 330 μL of a 2x concentrated culture were seeded on 55 mm unvented NGM plates. Seeded plates were left to dry and grow for 3 days at room temperature for experiments with mixed spores and vegetative cells, or overnight for experiments with vegetative cells only. To obtain spore-pure bacterial cultures, PXN21 *B. subtilis* bacteria were grown in Schaeffer’s sporulation medium (SSM) as previously described (Donato et al., 2017) (containing per liter: 8 g of Difco Bacto-nutrient broth, 10 mL of 10% w/v KCl, 10 mL of 1.2% w/v MgSO_4_·7H_2_O, ∼1.50 mL of 1 M NaOH up to pH 7.6, 1.0 mL of 1 M Ca(NO_3_)_2_, 1.0 mL of 0.010 M MnCl_2_, and 1.0 mL of 1 mM FeSO_4_). Briefly, bacteria were grown in SSM medium at 37 **°**C for 48 h. The culture was heat-treated for 20 min at 80 °C to kill vegetative cells and then spun down. To obtain pure spores, the heat-treated pelleted cells was treated three times with lysozyme (25 μg/mL; for 30 min at 37 **°**C), washed each time with cold deionised water and centrifuged until the culture consisted of only phase-bright spores. The efficiency of the purification was tested by the Schaeffer Fulton staining method.

#### Characterization of biofilm formation by *B. subtilis* strains

*B. subtilis* biofilms were grown on MSgg medium (5 mM potassium phosphate and 100 mM MOPS at pH 7.0 supplemented with 2 mM MgCl_2_, 700 μM CaCl_2_, 50 μM MnCl_2_, 50 μM FeCl_3,_ 1 μM ZnCl_2_, 2 μM thiamine, 0.5% glycerol, 0.5% glutamate) (Branda et al., 2001) solidified with 1.5% select agar (Invitrogen) at 30°C at the indicated time points. To set up a biofilm, a 3 mL aliquot of LB medium was inoculated with an individual colony taken from an overnight plate and grown at 37°C to an OD_600_ of 1. Then 5 μL of the culture was placed onto an MSgg plate which was incubated at 30°C for morphology and hydrophobicity studies. Images of colony biofilms were recorded using a Leica MZ16FA stereomicroscope. Biofilm hydrophobicity was determined by placing a 5 μl droplet of water on the upper surface of biofilms that had been grown for 48 hours at 30°C (Hobley et al., 2013). The water droplet was allowed to equilibrate for 5 minutes prior to imaging using a ThetaLite TL100 optical tensiometer (Biolin Scientific).

#### Experiments with killed bacteria

For dietary restriction (DR) with killed *E. coli* OP50 experiments, bacterial cultures were grown as previously described until an OD_600_ of 1. DR was induced by seeding 200 μL of a 1x concentrated culture on 55 mm unvented NGM plate and the same amount but twice concentrated (2x final) was used for normal growth conditions. *E. coli* culture was completely spread in the plates and left to dry and grow for 24 h or 48 h. Bacteria were killed by UV irradiation (Watson et al., 2014) (254 nm, 5 J/cm^2^), using a UV crosslinker (CL 508, Cleaver Scientific).

For the experiments with killed *B. subtilis* PXN21, bacterial cultures were grown as previously described until an OD_600_ of 1 to have only vegetative cells present. 200 μL of a 2x concentrated culture were completely spread on 55 cm unvented NGM + 0.5 mM arginine plate and left to dry and grow for only 24h. Bacteria were killed by a combination of UV irradiation (254 nm, 5 J/cm2) and antibiotics treatment (200 μg/mL kanamycin and 1mg/mL carbenicillin) for 3h before transferring the worms onto them (Smolentseva et al., 2017). In all the conditions, the efficiency of the killing protocol was tested by sampling and streaking the killed bacteria in LB agar plates and incubated overnight at 37°C.

#### Experiments with bacterial extracts

*B. subtilis* PXN21 was inoculated from a fresh colony into 1 L of LB and left to grow at 37°C and 220 rpm for 48 h. Under this condition, the cultures of this strain are very saturated and reach a final OD_600_ of 4-5. Bacteria were pelleted at 14000 rpm for 30 min at 4°C and the supernatant was separated from the pellet. The supernatant was consecutively filtered twice with 0.45 μm and 0.22 μm vacuum cellulose acetate filters to completely get rid of the cells. The pellet was washed twice with 250 mL of cold water and then once with 60% cold ethanol, centrifuged each time at 5000 rpm and 4°C and resuspended with vortex. The final clean pellet was resuspended in 100 mL of PBS and the bacteria were killed with 3 flash freeze-thaw cycles with liquid nitrogen/water bath at 60°C, followed by 1 h of incubation with lysozyme (25 μg/mL) on ice. Cells were finally disrupted by sonication using 5 cycles of 30 s at 20 Hz (MSE Soniprep 150), with incubation on ice for 30 s between cycles to avoid overheating. For both the supernatant and the cell lysate, 3 sequential organic extractions with 1:2 ratio of supernatant to diclorometane (DCM) and 1:10 of cell lysate and DCM were performed, respectively. The organic phases were dried separately to fully remove the DCM using an EZ-2 Elite personal evaporator (GeneVac), in the very low BP mode. The evaporator was maintained at 40°C throughout the process and 15 mL glass tubes rinsed clean with DCM were used for concentrating the organic phase. The final dry extracts were resuspended in 1 mL of ethyl acetate with vortex and kept it at −80°C afterward. Since we started with 1L of material, both extracts were considered to be 1000x concentrated. Appropriate dilutions from the concentrated stock were prepared in ethyl acetate, mixed in a glass falcon tube with 100 μl of water and spread on the top of *E. coli* seeded 35 mm plates. Ethyl acetate alone was added to *E. coli* as a vehicle-only control.

#### Nitric Oxide (NO) experiments

Freshly prepared NGM agar plates were placed open in a tissue culture hood for 30 min to dry and facilitate rapid absorption. Next, 50 μL of 2x OD_600_ = 1 bacterial culture was spread atop the plate, and then a freshly prepared solution of 200 mM NO donor MAHMA NONOate (Sigma) in water was applied to NGM agar plates to achieve a final concentration of 2 mM, and 4 mM. Immediately afterward, ∼70 synchronized L1s worms were quickly transferred to the plate.

This protocol was shown to be efficient to extend *C. elegans* lifespan, even though MAHMA NONOate has a very short half-life at pH 6 (∼1 min) (Gusarov et al., 2013). For control experiments, the NO donor was substituted with an equal amount of distilled water. For measurements of the effect of NO on α-synuclein aggregation, worms were moved to freshly prepared NO plates every day starting from L1 and scored at day 1 and 3 of adulthood.

#### Quantification of aggregation

NL5901 pkIs2386[P*unc-54*::α-synuclein::YFP + unc-119(+)] worms were anaesthetized using 50 mM Levamisole (Sigma) and high magnification (40x objective) z stack images of the head region were obtained by using a Zeiss Axio imager 2 microscope. Fluorescent spots bigger than 1 μm^2^, present in the region between the tip of head and the end of the pharyngeal bulb, were quantified manually, assisted by the Fiji analyze particle function applied to maximum intensity projections of the z stacks. To do so, background subtraction (rolling ball radius of 10 pixels) and adjustment of the threshold (automatic) were applied to the images before the analysis of the number of particles. The total area of the aggregates was extracted from the particle analysis with Fiji and the mean aggregates size per diet was simply calculated considering the total number of aggregates in the corresponding area. 72 hours after plating of the L1s was counted as day 1 adult. At least 25 worms were quantified per time point per condition. Each experiment was performed in triplicate, unless stated otherwise.

#### Locomotion analysis

Thrashing assays were performed as described before (Pujols et al., 2018), with some modifications. *C. elegans* NL5901 worms were synchronized as described above and were cultured at 20°C on *E. coli* OP50 strain until they reached L4 developmental stage. They were then either remained on OP50 diet or transferred to *B. subtilis* PXN21 (regular 3 days seeded protocol). Thrashing was assayed on days 1, 3, 5, 7 and 10 of adulthood. 5 animals were placed in a 40 μl drop of M9 buffer on an unseeded plate. Movies of thrashing worms were recorded for 3 min using the WormLab tracking platform (MBF Biosciences) at 7.5 frames/second. Waves per minute were obtained by analyzing the last minute of each video (allowing the animals to recover for 120 s after picking them into the drop). Average frequencies were determined every 0.4 s. Experiments were performed in duplicate. 10 videos per condition and 5 animals per video were analyzed (a total of 100 worms per condition).

#### Lifespan assays

Lifespan assays were performed at 20°C as previously described with modifications (Greer et al., 2007). Briefly, 200-250 synchronized L1s were placed on to corresponding food conditions and were, starting at d1Ad, transferred every 2 days onto fresh food and assessed for survival. Worms that failed to respond to the transfer process and repeated gentle prodding were declared dead and removed. Individuals that were missing or needed to be removed due to internal hatching were marked as censored. Experiments were performed in triplicate.

#### Quantification of life-traits

To determine developmental growth rates, 40-65 worms were mounted on a 3% w/v agarose pad in a drop of 50 mM levamisole and their developmental stages were assessed under compound microscope (DIC, 40X magnification) at exactly 48 h, 60 h, and 72 h after the synchronized L1s were placed on food. Individuals were staged as early L4, late L4, or adult by using the 9 stages of vulva development as reference points as described before (Mok et al., 2015). Stages L4.1 to L4.4 were considered as early L4, L4.5 to L4.9 considered as late L4 and young adult category was based on a fully formed vulva. For size measurements, worms were photographed at 10x and the images analyzed using Fiji (ImageJ). A segmented line was drawn along the center line of each the worm, quantified with the measure function, and then calibrated based on the scale bar. To assess the egg-laying rate and brood size, L4 worms were singled on to 10 separate plates per condition and transferred every 24 hours on to fresh plates until day 5 adult. The numbers of progeny resulting from each day of egg-laying were counted 2 days later.

#### Immunoblot analysis

Day 1 adult worms (∼4000) were rinsed with M9 + 0.01% Triton X-100, washed 3 times to remove bacteria, pelleted and resuspended in 400 μL of HEPES-based detergent buffer (50 mM HEPES pH 8, 0.2% v/v Triton x 100, 150 mM NaCl, 10 mM NaF, 5 mM DTT) + 1x Protease Inhibitor Mix (GE Healthcare 80-6501-23). Worms were centrifuged at 14000 rpm for 1 min, flash freeze-thawed 5 times with liquid nitrogen/water bath at 80°C and kept at −80°C. Worm pellets were disrupted mechanically using a TissueLyser II (QIAGEN) for 4 cycles of 40 s at 30 Hz, with 200ul of 0.7mm zirconia beads (Biospec). The lysates were centrifuged at 14000 rpm for 1 min and total amount of protein was quantified by Bradford assay (Bio-Rad). NuPAGE (4%–12%) Bis-Tris-gels (Invitrogen) were used to analyze α-synuclein (from 3 μg total protein) and β-actin (from 20 μg total protein) under denaturing conditions as previously described (Landré et al., 2017). Following transfer to nitrocellulose (PALL), membranes intended for α-synuclein analysis were fixed for 10 min using 4% PFA and washed 3 times with PBS containing 0.1% v/v tween-20 prior to blocking. The immunoblots were probed using anti-α-synuclein monoclonal antibody (BD Biosciences) 1:2000 and anti-β-actin (Sigma) 1:500 with appropriate HRP-labeled secondary antibodies (DAKO) at 1:2000. Bound antibodies where detected using ECL.

For the blots corresponding to the time course experiments, 50 worms (in duplicates) from day 1, day 3, day 5, day 7 and day 10 adults in the different diets were manually picked into 50 μl of M9 + 0.01% Triton X-100, washed 3 times to remove bacteria and resuspended in 4xLDS sample buffer supplemented with 10 mM dithiothreitol. Worms were flash freeze on dry ice, sonicated at 4°C for 10 cycles of 40 s at intensity II (Bioruptor® Plus) and boiled at 95°C for 10 min. The lysates were centrifuged at 14000 rpm for 1 min and around 2.5 ul of samples from day 1 adults to day 10 adults were loaded in NuPAGE (4%–12%) Bis-Tris-gels (Invitrogen) and transfer to nitrocellulose (PALL). Membranes were probed with 1:6000 of anti-β-tubulin monoclonal antibody (Sigma) to adjust the volumes manually. Tubulin was specifically selected for these blots because of the higher sensitivity versus actin for samples with low protein content. A second blot was performed by using the previously adjusted volumes for the samples and probed with both 1:6000 of the anti-β-tubulin antibody and 1:2000 of the anti-α-synuclein antibody.

Native protein analysis was carried out using NativePAGE 4%–16% Bis-Tris-gels (Invitrogen) loaded with 30 μg total protein in 1x NativePAGE sample buffer (Invitrogen) containing 0.5% w/v Coomassie Brilliant Blue G-250 per lane. The gel was run at 150V for 2 hours in 1x NativePAGE running buffer (minus G-250; Invitrogen). Proteins where subsequently transferred to nitrocellulose and α-synuclein was detected as described above.

#### Nematode RNA Sequencing

2000 young adult worms (approximately 50-55h after plating of the L1s) grown on *E. coli* OP50, *B. subtilis* PXN21 or a 1:1 mix of *E. coli*: *B. subtilis*, were collected and washed three times with M9 + 0.01% Triton X-100 buffer. The pellet was resuspended in 400 μl of RNA Lysis buffer (Quick-RNA Microprep Kit, Zymo Research) and worms were mechanically disrupted as previously described and kept at −80°C. Total RNA was extracted from the samples according to the manufacturer’s instructions. Three independent biological replicates were used for each experimental condition.

RNA samples were sent to Edinburgh Genomics for QC check and sequencing. Briefly, quality check of the samples was performed using Qubit with the broad range RNA kit (Thermo Fisher Scientific) and Tapestation 4200 with the RNA Screentape for eukaryotic RNA analysis (Agilent). Libraries were prepared from 5 μg of total RNA using the TruSeq Stranded mRNA kit (Illumina), and then validated. Samples were pooled to create 9 multiplexed DNA libraries, which were paired-end sequenced on an Illumina HiSeq 4000 platform. At least 290M + 290M 75 nt PE reads were obtained (one lane).

Sequence reads were trimmed using Cutadapt (version cutadapt-1.9.dev2; Martin, 2011) for quality at the 3′ end using a quality threshold of 30 and for adaptor sequences of the TruSeq stranded mRNA kit (AGATCGGAAGAGC), with a minimum length of 50. After trimming, reads were aligned against the *C. elegans* (WBcel235_ens8) genome from Ensembl release 84 with STAR) (version 2.5.2b; Dobin et al., 2013) with default parameters, except for specifying paired-end reads and the option “–outSAMtype BAM Unsorted.” Count tables for the different feature levels were obtained from bam files using the featureCounts (Liao et al., 2014) package version 1.5.3 with custom R scripts. Strandness was set to ‘reverse’ and a minimum alignment quality of 10 was specified. Gene names and other fields were derived from input annotation and added to the count/expression matrices. Count tables at the gene level presented a good correlation overall between replicates and samples.

Differential gene expression analysis was conducted for 17,708 genes whose expression was above a minimum “counts per million” (CPM) threshold level (CPM 0.1) in at least three samples. Differential gene expression was estimated using the edgeR package (Lun et al., 2016, Robinson et al., 2010) version 3.16.5 and resulting p values were adjusted using a false discovery rate (FDR) criterion. Genes with p values lower than 0.05, FDR values lower than 0.05 and a log2 fold change > 0.58 were considered to be differentially expressed. Heatmaps were generated using R with the pheatmap package (Kolde, 2015) (R package version 1.0.8.) or MeV (Multiple Experiment Viewer) version 4.9.0_r2731 (Howe et al., 2010).

Differential gene set analysis was carried out with the ROAST method (Wu et al., 2010) from the Limma package (version 3.30.13; Ritchie et al., 2015) of Bioconductor, using the same models and contrasts as used in differential expression. The following gene sets were used: Gene Ontology Biological Process, Molecular Function and Cellular Component downloaded from Ensembl version 91 (Ashburner et al., 2000, Subramanian et al., 2005); Reactome Pathways (Croft et al., 2014) (Reactome, downloaded January 2018). ROAST was executed using 9,999 rotations (randomizations). Each gene set was annotated with those genes individually differential (in the same direction as indicated for the gene set) to an unadjusted p value of 0.05. KEGG pathways were visualized utilizing the pathview package (Luo and Brouwer, 2013) version 1.24.0 of Bioconductor.

#### Reverse transcription and quantitative real-time PCR (qRT-PCR)

For quantitative real time PCR, 3 μg of total RNA and poly-T(V) (20 nt long) and random hexamers (6 nt long) were used for cDNA synthesis using SuperScript III Reverse Transcriptase (Thermo Fisher Scientific) and following manufacturer’s instructions. Quantitative PCR analyses were performed with 1:50 dilutions of the cDNAs using LightCycler 480 SYBR Green I Master mix and a LightCycler 480 II (Roche), following manufacturer’s instructions. Expression of all the genes was normalized to the geometric mean of *cdc-42* and F25B5.5 references genes. Expression of these genes was not variable between *E. coli* and *B. subtilis* in the RNaseq results. Data was analyzed by using the standard curve method and normalized to *E. coli* or *E. coli* arginine samples, respectively. For primer sequences see Table S9, all the primers were ordered from Sigma.

### Quantification and statistical analysis

All assays were performed in triplicate, unless stated otherwise. Graphs and statistical analysis were performed using Graphpad Prism 7. Data shown are presented as mean ± SEM. Statistical significance was calculated by unpaired t test, one-way or two-way ANOVA and Bonferroni multiple comparisons post hoc test, with p < 0.05 considered statistically significant. Statistical significance levels are denoted as follows: ^∗∗∗∗^p < 0.0001; ^∗∗∗^p < 0.001; ^∗∗^p < 0.01 and ^∗^p < 0.05. All statistical tests were two tailed where applicable.

For the expression levels by qRT-PCR, 3 independent biological samples with technical triplicates were analyzed by using two-way ANOVA with Bonferroni’s post hoc test of logNRQ.

ImageJ and Fiji were used to quantify the number and size of the aggregates as described above.

For the lifespan assay, Kaplan-Meier survival curves were generated using the statistical analysis software Graphpad Prism 7. Comparisons were made using the Log-rank (Mantel-Cox) test in both Graphpad Prism 7 and the online survival analysis tool OASIS 2 (Han et al., 2016).

For the immunoblots, quantification of the signals was performed using the gel analysis function of ImageJ and α-synuclein intensity was normalized against β-actin/tubulin signals.

Locomotor analysis was performed using WormLab tracking platform and software (MBF Biosciences).

### Data and code availability

The accession number for the *C. elegans* RNA sequencing data reported in this paper is ArrayExpress: E-MTAB-8164.

The authors declare that all data supporting the findings of this study are available within the article and its Supplementary Information files or upon request.

Code is also available either online where indicated or upon request.

## Supplemental Information

Document S1. Figures S1–S5 and Table S9

Table S1. Lifespan Assays, Related to Figure 2

Table S2. Differentially Expressed Genes in *B. subtilis* versus *E. coli*, Related to Figure 7

Table S3. Differentially Expressed Genes in Mix versus *E. coli*, Related to Figure 7

Table S4. Common Upregulated Genes in *B. subtilis* and Mix versus *E. coli*, Related to Figure 7

Table S5. Common Downregulated Genes in *B. subtilis* and Mix versus *E. coli*, Related to Figure 7

Table S6. Overlap between Differentially Expressed Genes and Previous Published Datasets, Related to Figure 7

Table S7. BP GO Term Analysis in *B. subtilis* and Mix versus *E. coli*, Related to Figure 7

Table S8. Expression Values of Genes from the KEGG Sphingolipid, Related to Figure 7

Document S2. Article plus Supplemental Information
