# Supplementary material for: Probiotic Bacillus subtilis Protects against α-Synuclein Aggregation in C. elegans
Source: Cell Rep. 2020 Jan 14;30(2):367–380.e7. doi: 10.1016/j.celrep.2019.12.078 (PMC6963774; doi:10.1016/j.celrep.2019.12.078)
Supplement: Document S1. Figures S1–S5 and Table S9 [file mmc1.pdf]

Cell Reports, Volume *30*

Supplemental Information

Probiotic *Bacillus subtilis*

Protects against α-Synuclein

Aggregation in *C. elegans*

María Eugenia Goya, Feng Xue, Cristina Sampedro-Torres-Quevedo, Sofia Arnaouteli, Lourdes Riquelme-Dominguez, Andrés Romanowski, Jack Brydon, Kathryn L. Ball, Nicola R. Stanley-Wall, and Maria Doitsidou

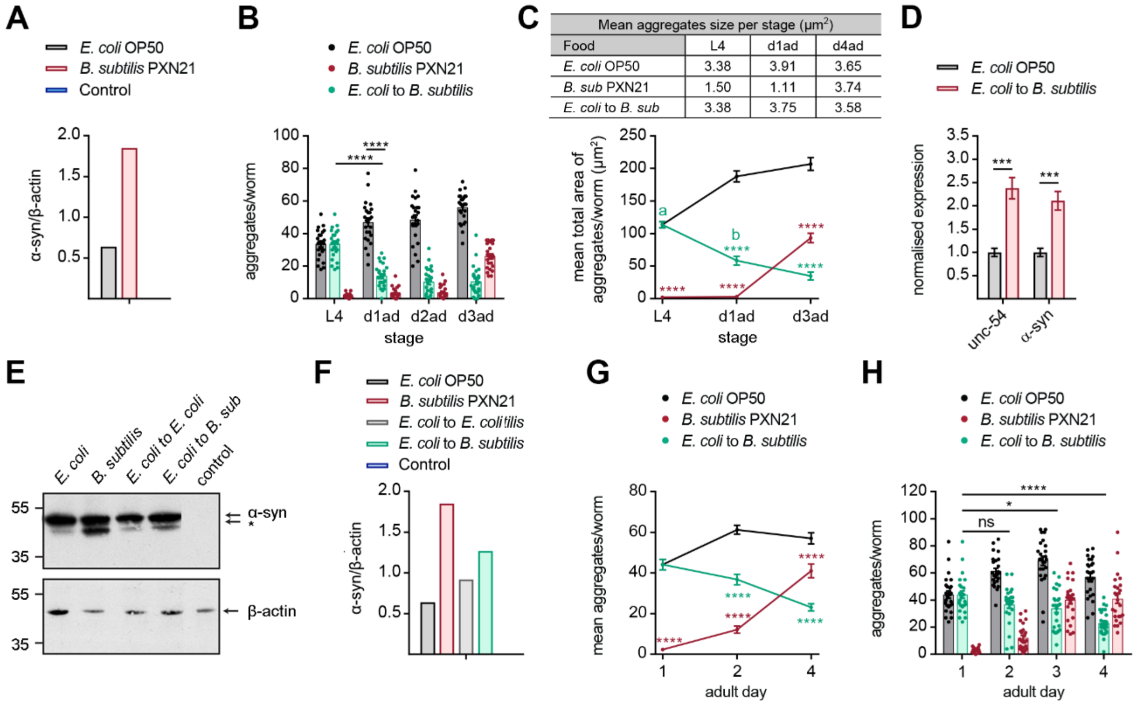

Figure S1 | *B. subtilis* PNX21 strain prevents and reverses α-synuclein aggregation in the *C. elegans* model NL5901 (P*unc-54*::α-syn::YFP). Related to Figure 1. (**A**) Normalised protein levels of α-syn vs β-actin signals shown in **Fig. 1D**. **(B)** Quantification of α-syn aggregates larger than 1 μm^2 per animal in the head region for the food-switching experiment (food switch at L4, see **Fig. 1G**). Most of the reversibility effect is reached 24 h after shifting to a *B. subtilis* PXN21 diet. ****P<0.0001 vs *E. coli;* a vs b, ****P<0.0001; n=25 worms per time point per condition. (**C**) Quantification of the total area of all aggregates larger than 1 μm^2 and the mean aggregates size per animal in the head region shown in **Fig. 1G**. (**D**) Expression levels by qRT-PCR of *unc-54* and α-syn transcripts from day 1 adult worms on *E. coli* or 24 h after switching to *B. subtilis* PXN21 mixed lawns, normalized to the *E. coli* diet. Expression level of each gene in *E. coli* was taken as 1. ***P<0.0004; n=3 per condition, with 3 technical replicates each (n represents a population of ~4000 worms). (**E**) SDS/PAGE from α-syn transgenic and wild type day 1 adult worms before and after the food-switching (food switch at L4, see **Fig. 1G**). Arrow with * indicates α-syn monomeric isoform. (**F**) Normalised protein levels of α-syn vs β-actin signals shown in **E**. **(G, H**) Quantification of α-syn aggregates in worms before and after food-switching at day 1 adult, from *E. coli* to *B. subtilis* PXN21. Aggregates progressively clear at 24 h, 48 h and 72 h after shifting to a *B. subtilis* PXN21 diet (**H**). ****P<0.0001 vs *E. coli* (**G**)*;* *P=0.015, ****P<0.0001 (**H**); n=25 worms per time point per condition. Data shown are mean ± SEM from one representative experiment out of three with similar results, unless stated otherwise. ns: no significant differences.

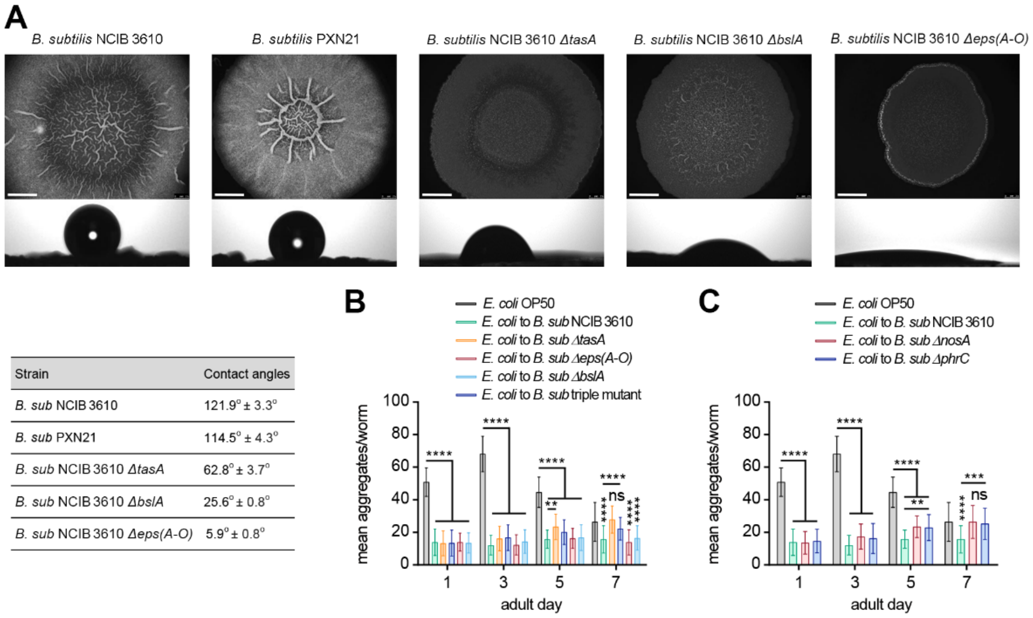

Strain	Contact angles
B. sub NCIB 3610	121.9° ± 3.3°
B. sub PXN21	114.5° ± 4.3°
B. sub NCIB 3610 *ΔtasA*	62.8° ± 3.7°
B. sub NCIB 3610 *ΔbslA*	25.6° ± 0.8°
B. sub NCIB 3610 *Δeps(A-O)*	5.9° ± 0.8°

Figure S2 | Biofilm formation and active metabolites contribute to the *B. subtilis* effect. Related to Figure 3. (**A**) Biofilm formation ability of *B. subtilis* PXN21 with respect to other well characterized *B. subtilis* NCIB 3160 strains. In the upper panels, representative *B. subtilis* strains in solid MSgg biofilm-inducing medium are shown. PXN21 is able to form highly complex and elaborate biofilm structures similar to the undomesticated NCIB 3610. These structures are severely disrupted in the isogenic NCIB 3610 deletion strains *ΔtasA, ΔbslA*, and *Δesp(A-O)*. Hydrophobicity tests (an important assessment of biofilm function), performed by adding a drop of water on top of the biofilm structure, are shown for each strain on the lower panels of each image and quantified on the table (bottom left). Biofilms with contact angles above 90° are consider hydrophobic. Scale bars=0.5 cm. Mean ± SEM, n=25 biofilm/strain from 5 independent experiments are shown. (**B)** Average α-syn aggregation of worms fed with *E. coli* or switched from *E. coli* to *B. subtilis* wild isolate NCBI 3610 or its matrix biofilm-deficient derivatives, *ΔtasA, ΔbslA*, *Δeps(A-O)* and the triple mutant (see **Fig. 3A**). ****P<0.0001, **P=0.0049; n≥25 worms per time point per condition. (**C**) Average α-syn aggregation in worms fed with either *E. coli*, switched from *E. coli* to *B. subtilis* NCBI 3610, or the NO and CSF deficient derivatives *ΔnosA* and *ΔphrC,* respectively (see **Fig. 3B**). ****P<0.0001, **P<0.01 and ***P=0.0001; n≥25 worms per time point per condition. Data shown are mean ± SEM from one representative experiment out of three with similar results, unless stated otherwise. ns: no significant differences.

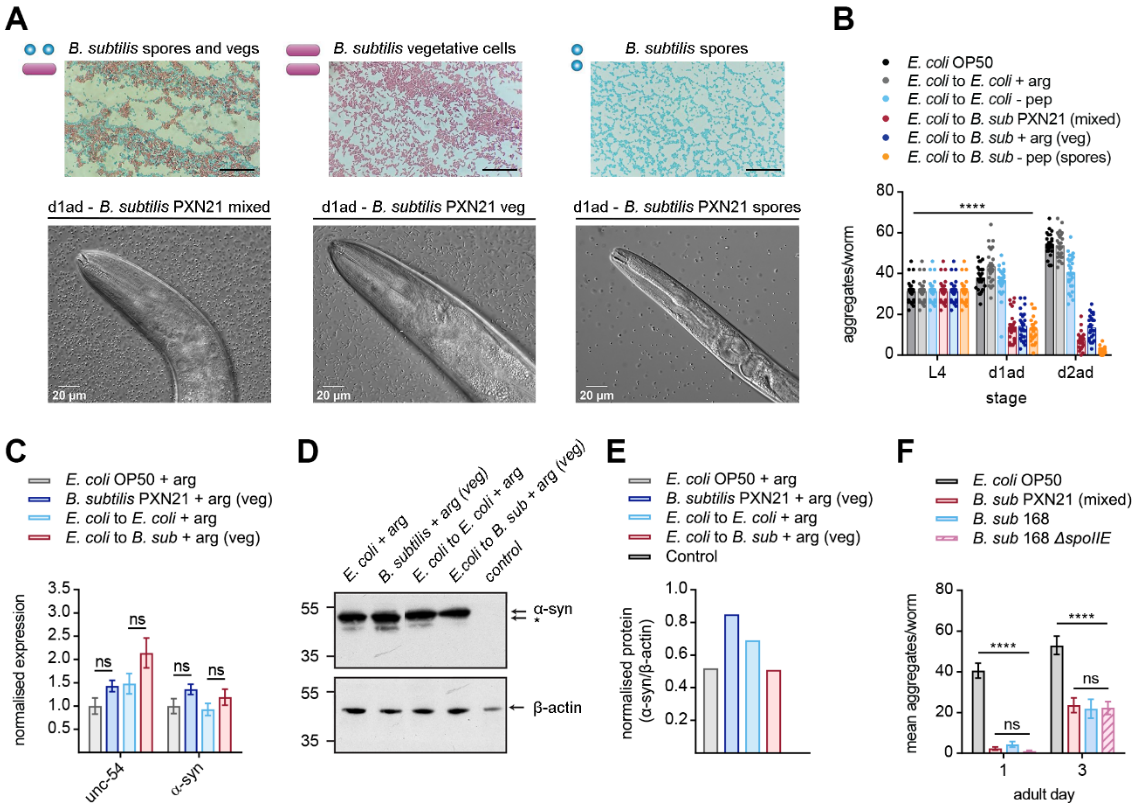

Figure S3 | *B. subtilis* spores and vegetative cells both protect against α-synuclein aggregation. Related to Figure 4. (**A**) Phenotypes of *B. subtilis* PXN21 tested in this work. Upper panels show Schaeffer Fulton staining of *B. subtilis* in different growing conditions. Scale bars=50 µm. In regular NGM medium and 3 days after seeding, *B. subtilis* forms a mix of spores (blue spheres) and vegetative cells (pink rods, left). In NGM supplemented with arginine (+ arg) and only 24 h after seeding, vegetative cells are predominant (middle). The spore-only condition is achieved by using a minimal NGM medium without peptone (- pep) and a short drying time (24 h) to prevent germination (upper right). Day 1 adult α-syn transgenic worms (fed from L1) with their corresponding diets are shown at the bottom. (**B**) Quantification of α-syn aggregates per animal in the head region for the food-switching experiment from *E. coli* to different *B. subtilis* PXN21 diets (see **Fig. 4C**). Most of the reversibility effect is reached only 24 h after shifting to any of the *B. subtilis* diets. ****P<0.0001; n=25 worms per time point per condition. (**C**) Expression levels by qRT-PCR of *unc-54* and α-syn transcripts from day 1 adult worms grown continuously on *E. coli* or *B. subtilis* PXN21 vegetative cells and after the switching to *B. subtilis* vegetative cells. Expression level of each gene was normalised to *E. coli* levels, which was taken as 1. n=3 per condition, with 3 technical replicates each (n represents a population of ~4000 worms). (**D**) SDS/PAGE from α-syn transgenic and wild type day 1 adult worms fed on a vegetative *B. subtilis* diet from L1 or L4, compared to *E. coli* (food switch at L4, expression levels shown in **D**). Arrow with * indicates α-syn monomeric isoform. (**E**) Normalised quantification of protein levels of α-syn vs β-actin signals shown in **D**. (**F)** Average number of α-syn aggregates in worms grown on *E. coli, B. subtilis* PNX21, *B. subtilis* 168, and its derivative *ΔspoIIE*. As shown in **Fig. 4D** for pure vegetative cells, in mixed lawns, wild type and *ΔspoIIE B. subtilis* 168 strains both protect from α-syn aggregation similarly to the PXN21 strain. n=50 worms per time point per condition. Data shown are mean ± SEM from one representative experiment out of three with similar results, unless stated otherwise. ns: no significant differences.

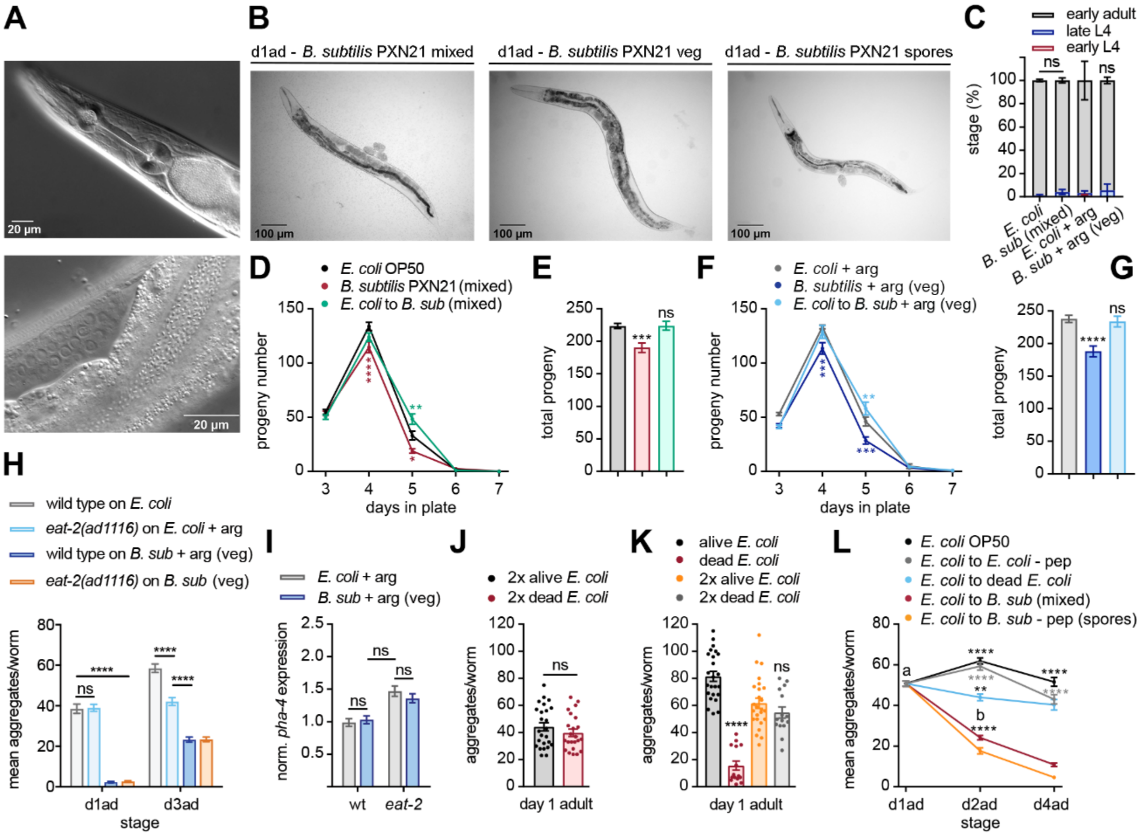

Figure S4 | *B. subtilis* reduces α-synuclein aggregation through dietary restriction dependent and independent mechanisms. Related to Figure 5. (A) Undigested spores visible in the gut of day 1 adult α-syn transgenic worms fed on *B. subtilis* PXN21 mixed cell-lawns (Nomarski images). **(B)** Day 1 adult worms grown on a *B. subtilis* PNX21 spores-only diet from L1 larval stage show severe signs of DR (strong developmental delay, very few eggs, and small size) (right) in comparison with animals fed on a mixed (left) or vegetative exclusive diet (middle). (**C**) Developmental stage at 60 h of α-syn-expressing worms grown on *E. coli* or *B. subtilis* mixed-cell lawns or vegetative cells (nearly 100% of worms have reached adult stage by 60 h). n≥100 worms per condition from two biological replicates are shown. (**D-G**) Quantification of brood size from L4 stage by day (**D**, **F**), or as total number (**E**, **G**), of worms fed with the different diets. (**D**, **F**) *P=0.0113, **P=0.0019, ****P<0.0001. (**E**, **G**) ***P=0.0009, ****P=0.0001. n≥34 worms per condition from two biological replicates are shown. (**H**) Average number of α-syn aggregates in wild type or *eat-2(ad456)* worms grown on *E. coli* or vegetative *B. subtilis* lawns. Note that *eat-2* mutant worms grown on *E. coli* supplemented with arginine no longer show reduced levels of aggregation in day 1 adults compared to wild type. ****P<0.0001; n=50 worms per time point per condition from two biological replicates are shown. (**I**) *pha-4* expression levels quantified by qRT-PCR and normalized to *E. coli* levels of worms grown on the diet conditions shown in **H**. *eat-2* or wild type worms grown on arginine supplemented plates show no *pha-4* upregulation in either bacterial diet compared to *E. coli*. The *pha-4* expression level in worms fed with *E. coli* was taken as 1. n=3 per condition, with 3 technical replicates each (n represents a population of ~4000 worms). (**J**) Quantification of α-syn aggregates in day 1 adult worms fed on 2x concentrated, alive or UV-killed *E. coli*, 48h after seeding. ****P<0.0001; n=25 worms per condition. (**K**) Quantification of α-syn aggregates in day 1 adult worms fed on different concentrations of alive or UV-killed *E. coli*, 24 h after seeding. ****P<0.0001,

n≥15 worms per condition. Worms grown on 2x concentrated UV-killed *E. coli* show no further reduction in aggregation compared to the diluted UV-killed, indicating that the protective effect of the less concentrated UV-killed *E. coli* is not due to reduced pathogenicity but due to dietary restriction. (**L**) Average α-syn aggregates of worms before and after day 1 adult switching to regular or dietary restriction inducing conditions. ****P<0.0001 indicates comparison of each diet vs its respective *E. coli* control; a vs b, **P=0.0026 for *E. coli* to UV-killed *E. coli* vs *E. coli*; ****P=0.0001 for *E. coli* to *B. subtilis* vs *E. coli*; n=25 worms per time point per condition. Data shown are mean ± SEM from one representative experiment out of three with similar results, unless stated otherwise. ns: no significant differences.

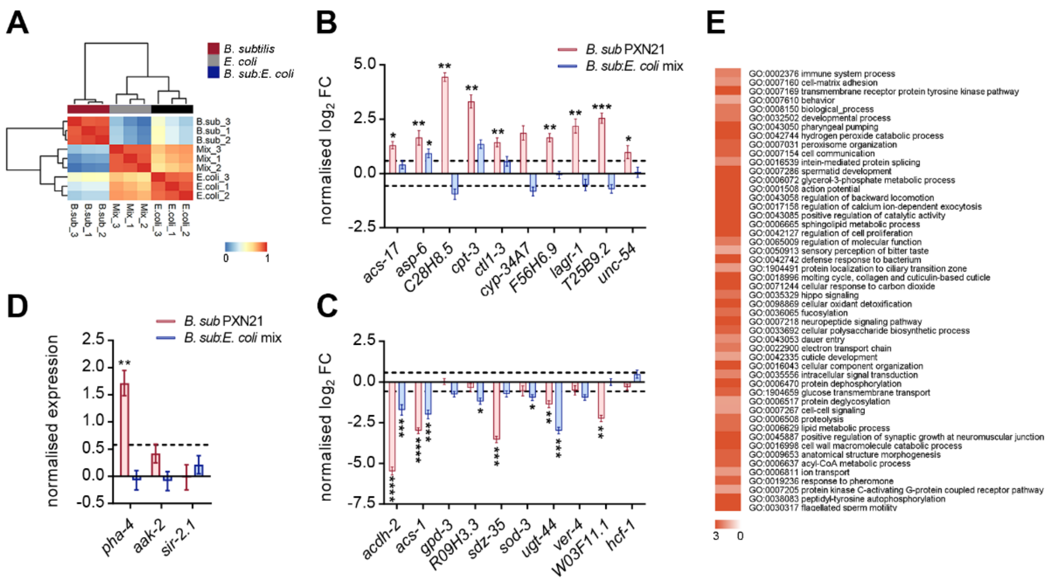

Gene	logFC (*B. sub* vs *E. coli*)	P value	FDR	logFC (mix vs *E. coli*)	P value	FDR
aak-2	0.3610	0.0001	0.0005	-0.0957	0.1360	0.2299
pha-4	0.4458	0.0038	0.0076	-0.0565	0.6461	0.7468
sir-2.1	-0.2885	0.0008	0.0020	0.2231	0.0001	0.0010

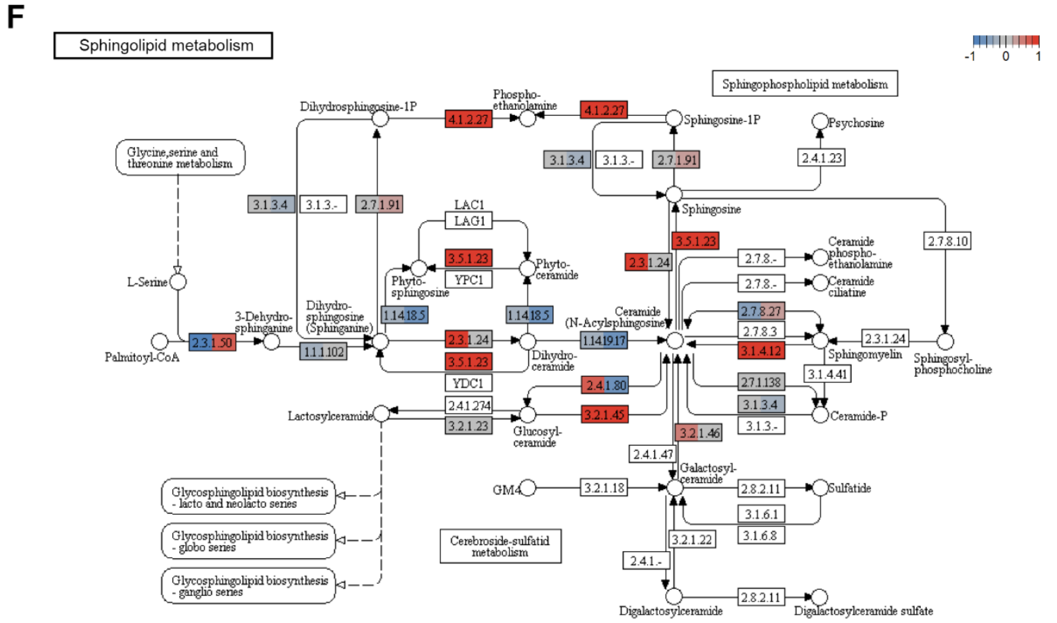

Figure S5 | *B. subtilis* protects against α-synuclein aggregation by changing the sphingolipid metabolism in the host. Related to Figure 7. (**A**) Heatmap showing the hierarchical clustering of samples by normalised expression values for all samples, generated by pheatmap. The 1:1 mixture of *B. subtilis* PXN21: *E. coli* OP50 shows a gene expression profile closer to *E. coli* than *B. subtilis*. (**B, C**) Normalised $\log_2$ fold-change expression levels of 10 random upregulated (**B**) and downregulated (**C**) genes from the RNAseq by qRT-PCR from α-syn-expressing young adult worms (approx. 50-52 h after hatching). Expression level of each gene in worms fed with *E. coli* was taken as 1. Dashed lines indicate the 1.5-fold change threshold ($\log_2$ FC 0.58 and -0.58). *P<0.05, **P<0.01 and **P<0.001; n=3 per condition, with 3 technical replicates each (n represents a population of ~4000 worms). (**D**) Normalised expression levels, as quantified by qRT-PCR, for a selection of dietary restriction transcription factors from young adult worms. Expression level of each gene in worms fed with *E. coli* was taken as 1. The bottom table shows the extracted values from the RNAseq analysis. **P=0.034; n=3 per condition, with 3

technical replicates each (n represents a population of ~4000 worms). (**E**) Summary of the top 50 non-redundant BP GO terms of *B. subtilis*:*E. coli* mix vs *E. coli* by $\log_{10}$ P value. (**F**) Visualization of *B. subtilis* and *B. subtilis*: *E. coli* mix vs *E. coli* beta scores over the sphingolipid pathway generated by Pathview. The left and right portions of a gene box represent *B. subtilis* and *B. subtilis:E. coli* beta scores, respectively. Red indicates a positive beta score, blue indicates a negative beta score, and grey marks genes that are neither positively nor negatively selected. White gene boxes correspond to genes that have not been found in the *C. elegans* genome. Data shown are mean ± SEM from one representative experiment out of three with similar results, unless stated otherwise. ns: no significant differences.

Table S9. | Oligonucleotides used in this study. Related to the STAR Methods.

PCR genotyping of *C. elegans* mutants	
**Primer name**	**Primer sequence**
daf16(mu86) Fw1	TCCGTCACAATCTGTCTCTTCA
daf16(mu86) Rv1	AAGTGTCGAGTGAAGGGAGC
daf16(mu86) Fw2	CGACAAGACAGGCGGTATCC
daf16(mu86) Rv2	TATCCTCTTCTTGGCTCCGC
daf2(e1370) Fw	GAGTCGCTCAAGTTTTGCCAT
daf2(e1370) Rv	CTCGACGTTCTCAACATCCG
hsf-1(sy441) Fw	CCGCAACAAGACTATTCGGG
hsf-1(sy441) Rw	ACAAATCCTCGGCTCCATCA
eat-2(ad465) Fw	TATGACCCAGTAGAACGGCC
eat-2(ad465) Rv	TGGAAGTAGTTGGTGGAGGG
lagr-1(gk331) Fw	AGGTGTCGGAGGTCGATG
lagr-1(gk331) Rv	AGTACCCGAATCGTTCTGG
sptl-3(ok1927) Fw1	AGGTTCTACAACATGGGTGG
sptl-3(ok1927) Rv1	GTTCCAGCAGAGTATCGACG
sptl-3(ok1927) Fw2	CTTCTCGCATCGATCTGGAG
sptl-3(ok1927) Rv2	TTGGGCTAACTCCACACAAC
asm-3(ok1744) Fw1	ACGACCCGGAGATTAGTAGG
asm-3(ok1744) Fw1	GACTTCGCGCGTAATTGAAG
asm-3(ok1744) Fw2	TGTGGACCCCATCAATTGTG
asm-3(ok1744) Rv2	GGGACACGGTGTGGTATAAC

PCR genotyping of bacterial mutants	
**Primer name**	**Primer sequence**
Universal16S-27Fseq-Fw	AGAGTTTGATCMTGGCTCAG
Universal16S-907Rseq-Rv	CCGTCAATTCMTTTRAGTTT
UP2-*B. subtilis* 168 library Fw	GAGGGAGGAAAGGCAGGA
UP3-*B. subtilis* 168 library-Rv	CGCCGTATCTGTGCTCTC
SpoIIE-B. subtilis 168 Fw	CGAAGATTTCTTTGGTATTG
SpoIIE-B. subtilis 168 Rv	AATGTCCGTTGTTCACATTCA
tasA-B. subtilis NCIB3610 Fw1	GGAAACAGATACAAAGGACAGC
tasA-B. subtilis NCIB3610 Rv1	CATCGAGACGGCCCAGTATATG
tasA-B. subtilis NCIB3610 Rv2	TCTTCTGGAGATGTATTGCTGC
eps(A-O)-B. subtilis NCIB3610 Fw	TCATTAACAGAAAGGGGCGC
eps(A-O)-B. subtilis NCIB3610 Rv1	AACACTAGTGCAGGACCAG
eps(A-O)-B. subtilis NCIB3610 Rv2	TTCCGAAGCCTTCGCCTC
bslA- B. subtilis NCIB3610 Fw	GTAAAGCAGAAAACGCCTGG
bslA- B. subtilis NCIB3610 Rv1	GTTCCCCCGTTCGTGTTTG
bslA- B. subtilis NCIB3610 Rv2	TCTGTGTTGTACGCAAGGC
phrC-B. subtilis NCIB3610 Fw	TCGAACGATGTTTCCAGTAC
phrC-B. subtilis NCIB3610 Rv	CTTGATCATTGTGGGAACTGC
nosA-B. subtilis NCIB3610 Fw	GTTTTCCAGGAAGTTGCAGA
nosA-B. subtilis NCIB3610 Rv	TGGCGGTTTCAATATGGTGA

Quantitative real time PCR (qRT-PCR)	
Primer name	Primer sequence
cdc-42 Fw	CTGCTGGACAGGAAGATTACG
cdc-42 Rv	CTCGGACATTCTCGAATGAAG
F25B5.5 Fw	GAAAGCAGTGTTCGACAATTCG
F25B5.5 Rv	CCTATGAGTTGCGCTTTCAATG
unc-54 Fw	AAGACCCAGAAGAAGCAGGTTG
unc-54 Rv	GATCGCATCTTTGAGAGGGAGT
alfasyn Fw	ATGTAGGCTCCAAAACCAAGGA
alfasyn Rv	TGCTCCTCCAACATTTGTCACT
pha-4 Fw	CGGCTGTTAATCACAGTCAACC
pha-4 Rv	GGTAAGGAGACGCGTATTGACC
sir-2.1 Fw	GTTCGTCTTGCTCATCAAATGC
sir-2.1 Rv	GCTCGTTGCAAGTCCAGATGTA
aak-2 Fw	GAGGCGAGTATTGAGAAAATGG
aak-2 Rv	AGTCTGGGAAAGCTTAGCTCCT
acs-17 Fw	GTAGAGGCATTGCAAAAGGAGT
acs-17 Fw	TTCTTTTGAGCTTCAAGGCTTC
asp-6 Fw	GATTCGGACCATCATGGATTCT
asp-6 Rv	CGAATCCCATTCTCTTGTTTCC
C28H8.5 Fw	CCGAATCAAGTGAAGTTTAGGC
C28H8.5 Rv	TCACGGAACATAATTCGAACAG
cpt-3 Fw	GTACAATAGCCCGTTCCTTGAC
cpt-3 Rv	CAATGGAGTTTGTCATGTTTGG
ctl1-3 Fw	CGACTGGAGATGTTGATCGTTA
ctl1-3 Rv	AGCACTTTCTCCCAGAACTGAC
cyp-34A7 Fw	GACGAGGAAACGTTCAAAAATC
cyp-34A7 Rv	TCAAATACAGCTCAGCTTTTGC
F56H6.9 Fw	CATGCAGCTATCTCTGGGTAGA
F56H6.9 Rv	TGCACAATATGTTTTCCTGTCC
lagr-1 Fw	GACAATCGTGAATGGGACACAA
lagr-1 Rv	GTTCTTCGGTTTAAGATGGCAC
T25B9.2 Fw	GCTGGATCTTCAAATGGTTATCCGC
T25B9.2 Rv	CAATACTAAACACAGCCGCTGTA
acdh-2 Fw	GGATCAAAATGGGGAATCAGTA
acdh-2 Rv	GATCTCGATCCACAATGAAACA
acs-11 Fw	GATATTAAGGCTTGGTGCAAGG
acs-11 Rv	CACTTTTCCAGTCACTGTCAGC
gpd-3 Fw	CACACTTTGTCAAGCTCGTCTC
gpd-3 Rv	TAGGCCTTGGTAGCAATGTAGG
R09H3.3 Fw	CAACTCTCAGCCATCGTACCAC
R09H3.3 Rv	GTCGGTTTCCTGTTGGTCTCTC
sdz-35 Fw	ACGAAACAATCCAACAAAATCC
sdz-35 Rv	TATCCTCCTCCAACTTTTCCAA
sod-3 Fw	ACCTTCAAAGGAGCTGATGGAC

sod-3 Rv	AGCCTTGAACCGCAATAGTGAT
ugt-44 Fw	AGATTTGGAAAACCACATGGAC
ugt-44 Rv	GCTCTTAGAACCTTCGAAACGA
ver-4 Fw	ACGTTCACACAAAAATCGGATG
ver-4 Rv	TCAAATACAGCTCAGCTTTTGC
W03F11.1 Fw	CAACGTAGCTGCGGAGAAG
W03F11.1 Rv	TTAACCTCATTTGGTGGGTAGG
hcf-1 Fw	ATTGCTGCAAGAAATGAAAAGG
hcf-1 Rv	AAACGAGCTCTCTTTTGCTGAC
